# Divergent neuronal DNA methylation patterns across human cortical development reveal critical periods and a unique role of CpH methylation

**DOI:** 10.1186/s13059-019-1805-1

**Published:** 2019-09-26

**Authors:** Amanda J. Price, Leonardo Collado-Torres, Nikolay A. Ivanov, Wei Xia, Emily E. Burke, Joo Heon Shin, Ran Tao, Liang Ma, Yankai Jia, Thomas M. Hyde, Joel E. Kleinman, Daniel R. Weinberger, Andrew E. Jaffe

**Affiliations:** 1 Lieber Institute for Brain Development, Johns Hopkins Medical Campus, 855 N Wolfe St, Ste 300, Baltimore, MD 21205 USA; 2 McKusick-Nathans Institute of Genetic Medicine, Johns Hopkins University School of Medicine (JHSOM), Baltimore, MD 21205 USA; 3 Center for Computational Biology, Johns Hopkins University, Baltimore, MD USA; 4 Department of Neurology, JHSOM, Baltimore, MD USA; 5 Department of Psychiatry and Behavioral Sciences, JHSOM, Baltimore, MD USA; 6 Department of Neuroscience, JHSOM, Baltimore, MD USA; 7 Department of Mental Health, Johns Hopkins Bloomberg School of Public Health (JHBSPH), 615 N Wolfe St, Baltimore, MD 21205 USA; 8 Department of Biostatistics, JHBSPH, 615 N Wolfe St, Baltimore, MD 21205 USA

**Keywords:** DNA methylation, Neurodevelopment, Gene expression, Non-CpG methylation

## Abstract

**Background:**

DNA methylation (DNAm) is a critical regulator of both development and cellular identity and shows unique patterns in neurons. To better characterize maturational changes in DNAm patterns in these cells, we profile the DNAm landscape at single-base resolution across the first two decades of human neocortical development in NeuN+ neurons using whole-genome bisulfite sequencing and compare them to non-neurons (primarily glia) and prenatal homogenate cortex.

**Results:**

We show that DNAm changes more dramatically during the first 5 years of postnatal life than during the entire remaining period. We further refine global patterns of increasingly divergent neuronal CpG and CpH methylation (mCpG and mCpH) into six developmental trajectories and find that in contrast to genome-wide patterns, neighboring mCpG and mCpH levels within these regions are highly correlated. We integrate paired RNA-seq data and identify putative regulation of hundreds of transcripts and their splicing events exclusively by mCpH levels, independently from mCpG levels, across this period. We finally explore the relationship between DNAm patterns and development of brain-related phenotypes and find enriched heritability for many phenotypes within identified DNAm features.

**Conclusions:**

By profiling DNAm changes in NeuN-sorted neurons over the span of human cortical development, we identify novel, dynamic regions of DNAm that would be masked in homogenate DNAm data; expand on the relationship between CpG methylation, CpH methylation, and gene expression; and find enrichment particularly for neuropsychiatric diseases in genomic regions with cell type-specific, developmentally dynamic DNAm patterns.

**Electronic supplementary material:**

The online version of this article (10.1186/s13059-019-1805-1) contains supplementary material, which is available to authorized users.

## Background

Neurons are unique cells that persist throughout the lifespan, accumulating programmed developmental changes and environmental experience that fine-tune neural circuitry in the brain. During development and maturation, neurons undergo precisely coordinated cascades of genetic regulation that combine with experience to shape the cellular output via progressive changes to the epigenome. DNA methylation (DNAm) is an integral facet of the epigenome that plays a role in establishing cell identity and developmental trajectories as well as adapting to experience via regulation of gene expression. Previous large-scale studies of DNAm across human brain development have identified widespread reconfiguration of the methylome during development, but have been limited to homogenate tissue [1] or have used microarray technologies [2], creating ambiguity about the extent of cell type-specific developmental DNAm changes and effects on transcript isoforms across the genome [3]. Although cell type-specific DNAm patterns can be inferred for genes with known cell type-specific expression patterns, beginning an analysis with cell type-resolved DNAm data would facilitate the identification of DNAm remodeling outside that context.

To better characterize the DNAm landscape across human cortical development, we performed whole-genome bisulfite sequencing (WGBS, see the “Methods” section) on homogenate tissue and on a neuron-enriched population isolated from 24 human dorsolateral prefrontal cortex (DLPFC) samples aged 0–23 years using NeuN-based fluorescence-activated nuclear sorting (FANS, Additional file 1: Figure S1A). To complement these data, we sequenced 8 FANS-derived NeuN− postnatal samples and 20 homogenate prenatal cortical samples, for a total of 75 samples after quality control (Additional file 2: Table S1). We fully characterized the landscape of DNAm at both CpG and non-CpG (CpH) dinucleotides in these samples, allowing for a finer dissection of differential DNAm functional specificity. We also sequenced matched transcriptomes of homogenate cortical samples from these donors and a subset of 3 nuclear transcriptomes each from NeuN+ and NeuN− samples to assess the functional consequences of epigenomic remodeling (53 total transcriptomes, Additional file 2: Table S2). By exploring DNAm patterns in neurons across prenatal and postnatal human brain development, we show that the first 5 years of postnatal life are a critical period in epigenetic plasticity, and we identify developmental shifts in neuronal DNAm in both the CpG and CpH contexts. We also clarify the relationship between CpG but particularly CpH methylation (mCpG and mCpH, respectively) and gene expression and splicing in neuronal development and explore the ramifications of these insights for neuropsychiatric disease.

## Results

After data processing, quality control, and filtering, we analyzed 18.7 million cytosines in the CpG context at an average coverage of 15× (see the “Methods” section). Comparable to previous reports [1, 4, 5], CpGs were overall highly methylated (71–76% CpGs with *β* > 80%, Additional file 2: Table S3).

While NeuN antibody labels most mature neuronal subtypes in the human cortex, some neurons will not be labeled and will be captured in the NeuN− fraction amidst a diverse array of non-neuronal cell types, including oligodendrocytes, astrocytes, microglia, and epithelial cells. Gene expression differences between fractions confirmed, however, that NeuN+ and NeuN− samples are enriched for neuronal and glial lineage cells, respectively (Additional file 1: Figure S1B-D). Therefore, in this work, we refer to NeuN+ and NeuN− samples as “neurons” and “glia,” respectively, although we acknowledge that these samples do not perfectly reflect these identities and mask more granular differences between subcellular identities contained within.

Developmental DNAm changes identified in homogenate cortex were strongly confounded by shifting cell type proportions (OR = 7.5, *p* < 10^−100^, Additional file 1: Figure S2A) [2]. While homogenate measurements were positively correlated with developmental changes that occurred in both neuronal and glial cell types (*⍴* = 0.79, *p* < 10^−100^), cell type-specific developmental changes were less consistently observed in homogenate preparations (*⍴* = − 0.26, *p* < 10^−100^, Additional file 1: Figure S2B-D). Overall, ~ 40% of cell type-specific developmental DNAm changes could not be detected at all in the homogenate cortex (Additional file 1: Figure S2E), and many of the cell type-specific effects could not be accurately identified in homogenate tissue. These results highlight the importance of measuring DNAm in the appropriate cellular context for improved resolution to detect true developmental changes.

### DNAm as a map of putative functional genomic states

Local CpG methylation (mCpG) patterns are known to distinguish genomic states of DNA and chromatin. For instance, unmethylated regions (UMRs) are associated with promoters, with a subset of longer UMRs (DNAm valleys, DMVs) that overlap developmental genes often encoding transcription factors (TFs) [6, 7]; low-methylated regions (LMRs) often signify enhancer sequence [8]; and partially methylated domains (PMDs) are associated with heterochromatin and late replicating DNA [9–11]. To better resolve the developing regulatory landscape in postnatal neurons and glia and in bulk prenatal cortex, we assessed the temporal dynamics of these selected DNAm patterns in the CpG context. Compared to prenatal homogenate cortex and postnatal glial cells, postnatal neurons showed a general accumulation of mCpG, at a rate 50% faster than the other cells. This was evident in the LMR and to a lesser extent the UMR landscape, since fewer and smaller LMRs were identified as neuronal development progressed (Additional file 1: Figure S3A-B; Additional file 2: Table S4). As expected, UMRs and LMRs were highly enriched for transcription start sites (TSSs) and enhancers in DLPFC chromatin state data from the Roadmap Epigenomics Consortium [12] (Additional file 1: Figure S3C). Interestingly, LMRs were similarly enriched in these states in both adult and fetal brain; this correspondence may reflect a shared regulatory landscape established early in the development.

While PMDs are a common feature of most cell types, they have not been conclusively identified in neurons. Here, we identified a range of 245 to 404 PMDs per neuronal sample (Additional file 1: Figure S4A). PMDs were especially enriched for heterochromatin and, interestingly, enhancers in our postnatal neuronal samples (Additional file 1: Figure S4B). 65.4% of PMD base pairs were also identified as PMD in an independent WGBS dataset of NeuN-sorted human neurons (Additional file 1: Figure S4C). 40.3–61.0% of PMD bases per neuronal sample were identified as common PMD sequence, and 9.3–15.0% bases were additionally identified as PMD in at least one sample in a recent study profiling PMDs in multiple cell types and tissues [13] (Additional file 1: Figure S4D). These data suggest that although the neuronal genome was overall highly methylated, a small but consistent portion displayed the characteristics of PMDs.

We further identified significant neuronal DMV changes through the accumulation of mCpG that revealed regulators of cell identity and development and their temporal windows of expression change. Compared to the bulk prenatal cortex, postnatal neurons and glia showed a marked reduction in the size of DMVs (Additional file 1: Figure S5A). Although most transcription factor genes within DMVs were shared, methylation shifts within DMVs across the timespan studied led to the inclusion and exclusion of several genes, and on average, transcription factor genes were higher expressed in the age group in which the gene was escaping the DMV state by accumulating DNAm (Additional file 1: Figure S5B-C). These results underscore the substantial DNAm landscape alterations that neurons and glia undergo during development in defined mCpG patterns, including previously unobserved PMDs.

### Developmental shifts in neuronal mCpG highlight synaptic remodeling during the first 5 years of postnatal life

We next quantified more localized changing mCpG levels by exploiting the correlation between neighboring mCpG levels to identify the genomic regions with differential mCpG levels. We identified 11,179 differentially methylated regions (DMRs, FWER< 5%, see the “Methods” section) in the CpG context between cell types (covering 31.1 Mb) that replicated in independent WGBS data [1] (98.4% concordant, *p* = 0.925, Additional file 1: Figure S6A). Many of these DMRs overlapped genes involved in neuronal or glial-specific processes (Additional file 1: Figure S6B). We found fewer DMRs for developmental mCpG changes compared with the cell type differences, the majority being within rather than across cell types (2178 vs 129 DMRs, at ~ 5% change in DNAm per decade of life, FWER< 5%).

**Fig. 1:**
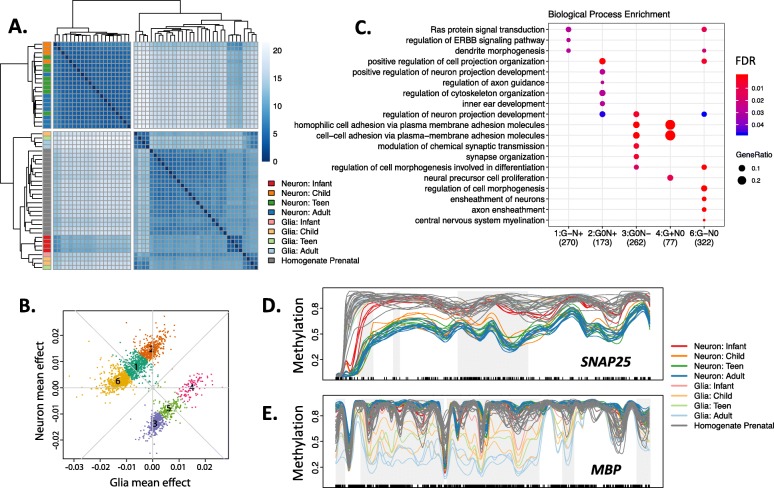
Regional cell type-specific developmental mCpG trajectories. **a** Euclidean distances between the samples within cdDMRs show that older neuronal samples cluster separately from infant neuronal samples, glia regardless of age, and bulk prenatal cortex. **b** Decomposing cdDMR patterns into six clusters using *k*-means based on glia and neuron mean mCpG changes per year of life. **c** The top five most enriched Gene Ontology terms for each of the six groups in **b** highlight diverse biological processes among the groups. No terms were enriched for group 5. **d** Example of group 3 cdDMR within *SNAP25.* **e** Example of group 6 cdDMR within *MBP.* Gray shading indicates the boundaries of the cdDMR, and black tick marks on the *x*-axis indicate the position of CpGs. Key: neuron (NeuN+), glia (NeuN−), infant (0–1 year), child (1–10 years), teen (11–17 years), and adult (18+ years)

Among the 2178 cell type-specific developmental DMRs (cdDMRs, 3 Mb, Additional file 2: Table S5), neuronal mCpG patterns seemed to diverge from an immature landscape shared by the glia and prenatal cortex (Fig. 1a), with the largest changes occurring in the first 5 years of life (Additional file 1: Figure S6C). Indeed, the magnitude of DNAm changes in neurons and glia in the samples 5 years and younger was double that of older samples ( file 1: Figure S6D-E). These results provide epigenetic correlates to the known developmental processes occurring in the cortex in the first five postnatal years, including prolific synaptogenesis and gliogenesis.

We further parsed these cdDMRs using *k*-means clustering to partition the cdDMRs into six groups with unique DNAm characteristics (Fig. 1b). 71.1% of cdDMRs were in groups characterized by increasing neuronal and/or decreasing glial DNAm over postnatal development (Fig. 1b; groups 1, 2, and 6). A varying proportion of each cdDMR group corresponded to the sequence differentially methylated by neuronal subtype from publicly available data [14] depending on the trajectory of neuronal methylation patterns in the group, suggesting that assorted neuronal subclasses contribute to these developmental patterns (Additional file 1: Figure S7A-B). Gene ontology enrichment in the six groups suggested that these groups are associated with a continuum of biological roles, many relating to the functions specific to the cell type with decreasing methylation (Fig. 1c). For example, Fig. 1d shows a group 3 cdDMR within *SNAP25*, a presynaptic neuronal gene, in which neurons uniquely and progressively lost DNAm over development. This pattern suggests increased repression of neuronal fate in maturing glia not mirrored in neurons over postnatal development in group 3 cdDMRs. Likewise, the opposite pattern was observed in a group 6 cdDMR within *MBP*, an oligodendrocyte gene encoding a component of the myelin sheath, in which glia but not neurons progressively lost DNAm (Fig. 1e).

We lastly compared these cdDMR groups to a list of putative enhancers active in human brain development curated by evolutionary age [15] and found strong enrichment for these sequences across all six groups (Additional file 1: Figure S8A). Human accelerated regions, or conserved sequences that have experienced rapid mutation in the human lineage [16], were also enriched for dynamic DNAm remodeling (Additional file 1: Figure S8B), suggesting that our CpG-based cdDMRs may be enriched for sequences related to higher cognitive functions associated with the human DLPFC.

Overlapping cdDMRs with the mCpG features identified above provided additional insight to the potential functional genomic states underlying these regions. For instance, cdDMRs scarcely overlapped heterochromatic PMDs; cdDMRs losing neuronal mCpG were positively correlated with increasing LMR overlap, potentially reflecting enhancer element activation during cortical maturation in these groups (groups 3 and 5 cdDMRs; both with *t* > 3.8, *p* > 0.63, and FDR < 2.7e−03). Curiously, a high proportion of cdDMRs gaining DNAm in glia but not in neurons (group 4 cdDMRs) overlapped DMVs early in the development in glia but steadily lost DMV status over time (*t* = − 4.3, *p* = − 0.87, FDR = 1.3e−02, Additional file 1: Figure S9A). Assessing chromatin state from the homogenate Roadmap Epigenomics brain maps, in contrast, lacked the resolution to provide this nuance: all six cdDMR groups were similarly enriched for transcriptional (particularly TSS-flanking) and enhancer chromatin states and depleted for heterochromatin and quiescent states (Additional file 1: Figure S9B). These results confirm the role of dynamic DNAm in helping establish epigenomic states that guide cell lineage differentiation and emphasize the utility of creating genome-wide DNAm maps to better parse the functional diversity of cell type-specific developmental DNAm remodeling in the human cortex, a process that is particularly critical during the first 5 years of postnatal development.

### Abundant neuronal CpH methylation is highly correlated with neighboring CpG methylation

Unlike in most other somatic tissues and cell types, mCpH is an abundant, conserved feature of the neuronal epigenome [1, 4]. We therefore analyzed 58.1 million cytosines in CpH contexts (H = A, T, or C) that had evidence of methylation across the samples (coverage ≥ 5, at least 5 samples with *β* > 0, see the “Methods” section). As shown previously [1], mCpH sites were predominantly lowly methylated (92–99% CpHs with *β* < 20%, Additional file 2: Table S3). While mCpH was distributed throughout the genome (Additional file 1: Figure S10A), it was greater in neurons than in glia (98.9% of 7,682,075 differentially methylated CpHs between cell types were in hypermethylated neurons at FDR < 5%) and mostly accumulated across postnatal development (99.3% of 3,194,618 CpHs, at FDR < 5%; Additional file 2: Table S6). Most mCpH accumulated primarily in either the CAG or CAC context over the first 5 years of postnatal life—similarly to mCpG—followed by a tapered global increase into adulthood (Additional file 1: Figure S10B-C).

**Fig. 2:**
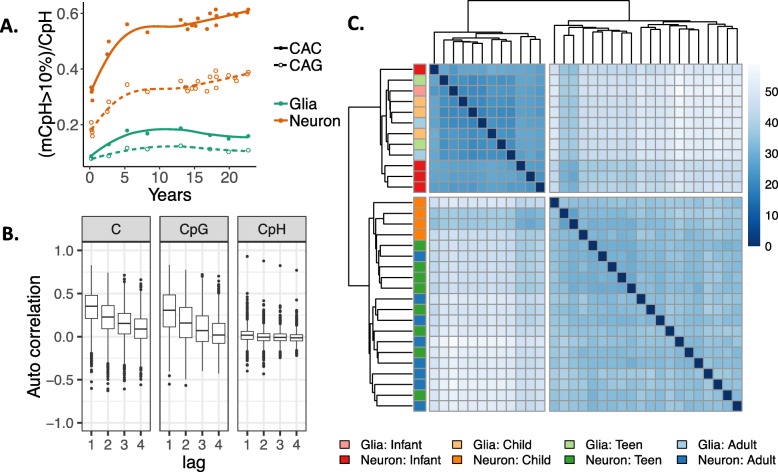
CpH methylation patterns across brain development. **a** The proportion of CAC and CAG sites that are greater than 10% methylated in neurons and non-neurons (glia) across brain development. The *y*-axis reflects the number of > 10% methylated CAC or CAG sites divided by the number of CAH sites in that trinucleotide context. **b** Autocorrelation levels for different cytosine contexts in neurons. Autocorrelation levels were similar for mCpG and all cytosines, with uncorrelated levels in the CpH context. **c** Euclidean distances between samples based on mCpH within cdDMRs again cluster infant neurons (dark red) with glia of all ages (light colors) rather than with older neurons

While the majority of mCpH in embryonic stem cells (ESCs) occur in the CAG context, previous work has shown that ESCs undergo loss of mCAG during neuronal differentiation followed by preferential accumulation of mCAC [17]. Here, we further refined these patterns and found a cell type-specific relationship with trinucleotide context: overall, total mCAG increased 40% faster than mCAC in neurons, while in glia, mCAG accumulated 50% slower than mCAC (Additional file 1: Figure S10C). Taking into account the relative genome-wide proportions of CAG and CAC though, neuronal mCAG accumulated 30% slower than mCAC (Fig. 2a). mCpH that was greater in glia than in neurons, or in younger than in older neurons, was more likely to be in the CAG than CAC context (OR > 4.13, *p* < 2.2e−16). Interestingly, the 3286 and 1744 genes that contained significantly increasing and decreasing mCAC vs mCAG over development, respectively, were associated with different biological processes related to neuronal function and activity, particularly involving the synapse (Additional file 1: Figure S10D). These results reinforce that methylation of different trinucleotide contexts may be regulated by distinct mechanisms playing non-redundant biological roles in human brain development.

We next examined the relationship between neighboring mC levels by measuring autocorrelation, defined as how correlated the methylation level of a cytosine is with that of cytosines progressively further away. Unlike in the CpG context, where neighboring mCpG levels were highly correlated as previously described [18], neighboring CpH DNAm levels across the genome were not autocorrelated. Within the cdDMRs, however, while mCpH levels separately remained uncorrelated, together all methylated cytosines (i.e., mCpH+mCpG) showed similar autocorrelation as mCpG levels alone (Fig. 2b). This was especially surprising given that there were about two times as many CpHs than CpGs within these regions and that the CpG and CpH were relatively interspersed, suggesting potential functional convergence in the developmentally regulated patterns identified by mCpG in these regions. Indeed, unsupervised hierarchical clustering of CpH within the cdDMRs showed infant neuronal mCpH levels were even more similar to glia compared to older neurons than mCpG (Fig. 2c). Examining the mean mCpH compared to mCpG within the *k*-means cdDMR clusters showed that the groups gaining mCpG were the most correlated with mCpH trajectories within the cdDMRs (*p* = 0.97, *t* = 17.6, *p* = 2.0e−14) and that although mCpG (unlike mCpH) is present at high levels prenatally, both mCpG and mCpH accumulate at similar rates over postnatal development in these groups, once again especially in the first 5 years of postnatal life where the majority of the methylation change takes place (*t* = − 0.091, *p* = 0.94; Additional file 1: Figure S6C and Additional file 1: Figure S11). These results emphasize the potential regulatory importance of cdDMRs and putative functional agreement between both contexts of DNAm in these regions.

### mCpG and mCpH levels influence transcript isoform use

Previous studies show that both mCpG and mCpH in gene bodies but particularly in the promoter and first 2 kb of the gene are negatively associated with gene expression and that genic mCpH is the most discriminating predictor of gene expression [1, 5]. To anchor our DNAm patterns in transcriptional activity, we compared our WGBS data with NeuN-sorted nuclear RNA-seq data (see the “Methods” section). We took the average DNAm levels across the six groups—infant (ages 0–1), child (ages 1–10), and teen (age 10+) within both cell types (neuronal and glial)—and calculated the associations between DNAm and expression. Gene expression was negatively correlated with mCpG levels regardless of age and cell type in both promoter sequence and gene bodies (− 0.42 < *p* < − 0.22, *p* ~ 0; 57,332 genes, *p* < 10^−100^; Additional file 1: Figure S12A). Interestingly, mCpG in exons was significantly but weakly positively correlated with exon expression in infancy, particularly in glial samples (*p* = 0.094, *p* < 10^−100^), which may relate to the previously identified positive relationship between mCpG and expression and higher methylation in exons than introns [9].

Across promoters, gene bodies, and exons, neurons showed a negative correlation between gene expression and mCpH that became stronger over the development, while glial samples showed a much weaker and developmentally consistent negative correlation (Additional file 1: Figure S12B). This pattern was consistent with the preferential accumulation of mCpH in neurons as the brain matures. mCAC and mCAG showed similar patterns of increasingly strong negative correlation preferentially in neurons between methylation and expression across these features (Additional file 1: Figure S12C-D). Both mCpG and mCpH surrounding the exon-exon splice junctions were weakly negatively correlated with the expression of the junction in neurons (Additional file 1: Figure S12E).

**Fig. 3:**
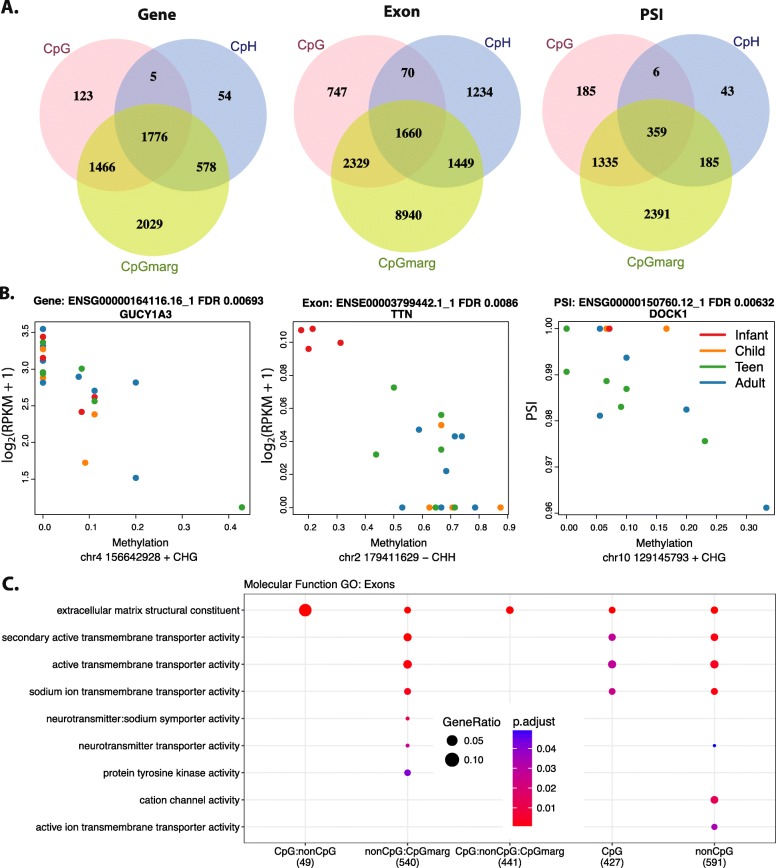
Methylation associations with expression. **a** Venn diagrams of the methylation associations by unique feature for the gene, exon, and PSI. The sets are determined by if the association is FDR < 5% genome-wide for CpG and CpH or if it is a CpG marginally significant within ± 1-kb window of a CpH association. **b** Example associations between methylation and expression at the gene level colored by age: red, infant; orange, child; green, teen; blue, adult. *GUCY1A3* contains one of the top CpH differentially expressed between neurons and glia. Expression of an exon of *TTN*, an autism-associated gene, is negatively associated with mCpH. *DOCK1* PSI of an alternative end site is negatively associated with mCpH. **c** Enriched molecular function ontology terms for methylation-associated exons by the Venn diagram groups from **a**

Because mCpG has previously been associated with alternative splicing [19] and mCpH is 15–20% greater in exons than in introns [9], we hypothesized that accumulating mCpH may contribute to the diversity of alternative splicing characteristic of the brain particularly during development. Leveraging our single-base resolution data, we were able to identify genome-wide functional correlates of mCpH, independent of nearby mCpG, by associating DNAm with nearby expression in the same cortical samples. Specifically, we tested whether methylation levels directly associated with gene or exon expression levels as well as the “percent spliced in” (PSI) of alternative splicing events using the 22 neuronal samples with matching homogenate polyA+ RNA-seq data (see the “Methods” section) [20]. We found 40,940 CpG and 40,303 CpH associations that explain changes in these three expression summarizations at FDR < 5% with a genome-wide *p* < 5 × 10^−4^. We further identified 220,622 marginal (*p* < 0.01) CpG associations with expression within 1 kb around the associated CpH. While an independent association of mCpH at the gene and PSI summarizations was rare, there were substantially more exons exclusively regulated by local mCpH, largely in the CHH context, in developing postnatal neurons (Fig. 3a). Three examples of methylation-associated isoform changes are shown in Fig. 3b.

Regardless of the context specificity, these expression-associated cytosines were depleted in gene promoters and instead enriched in the gene bodies and flanking regions (Table 1, see the “Methods” section). Both contexts were enriched for the high-GC 3′ and 5′ canonical splice site sequences (FDR < 1.1e−04), although the associated cytosine could be either inside or outside the corresponding expression feature. Only 3.5–13.7% of the expression-associated cytosines overlapped DMR sequence after stratifying by expressed feature and dinucleotide context, indicating that these associations may arise from a more individualized mC effect than the DMRs.

**Table 1:** Summary of methylation vs expression changes across feature types

Feature	Meth. type	Direction	*N*	*N* unique C	*N* unique features	Mean *N* Meth. > 0	Mean *N* Meth. < 1	Mean Expr change	Prop. age confounded FDR ≥ 5%	Prop. in promoter	Prop. in gene body	Prop. in gene flanking	Prop. CHG	Prop. CHH	Prop. DE glia	Prop. DE neuron	Prop. No Diff
Gene	CpG	Down	18,458	18,353	2074	21.9	21.9	1.39	0.0932	0.0671	0.966	0.906	0	0	0.126	0.329	0.546
		Up	5217	5021	1743	21.8	21.8	1.69	0.0845	0.0571	0.979	0.92	0	0	0.304	0.193	0.503
	CpH	Down	25,812	25,712	1611	21.1	21.1	1.4	0.115	0.0161	0.519	0.75	0.22	0.781	0.0912	0.413	0.496
		Up	3842	3840	846	20.1	20.1	1.66	0.2	0.0219	0.532	0.807	0.26	0.744	0.123	0.27	0.607
Exon	CpG	Down	9505	4536	4168	21.9	21.9	1.23	0.104	0.19	0.934	0.991	0	0	0.0097	0.0256	0.965
		Up	1328	638	687	21	21	1.93	0.191	0.175	0.928	0.988	0	0	0.0369	0.0136	0.95
	CpH	Down	7874	4082	3458	21.1	21.1	1.72	0.074	0.0328	0.437	0.927	0.19	0.814	0.0086	0.0451	0.946
		Up	1288	733	962	18.7	18.7	1.94	0.154	0.0435	0.481	0.918	0.28	0.722	0.0031	0.0311	0.966
PSI	CpG	Down	4286	2477	1315	21.7	21.7	0.124	0.0272	0.0434	0.994	0.936	0	0	0.242	0.212	0.547
		Up	5782	3915	1646	21.8	21.8	0.121	0.0195	0.0398	0.991	0.919	0	0	0.261	0.222	0.517
	CpH	Down	1296	945	489	16.8	16.8	0.447	0.0201	0.0274	0.537	0.676	0.36	0.644	0.187	0.286	0.526
		Up	875	536	391	16.5	16.5	0.169	0.032	0.0279	0.559	0.775	0.36	0.637	0.194	0.33	0.477

Mean values are shown for the number of samples with *β* > 0, *β* < 1, and expression change variables (either ΔPSI or Δlog2(FPKM + 1) for genes and exons). Columns from “Age confounded” are proportions. See Additional file 2: Table S12 for a full description of the variables

Although the majority of these DNAm-expression associations were independent from development despite being identified in developing neurons, the mCpH changes at these sites were independently associated with age and expression. The genes including PSI events regulated by local DNAm levels in both CpG and CpH contexts were consistently enriched for neuronal components (Additional file 1: Figure S13), while genes containing methylation-associated alternative exons were enriched for synaptic signaling and neurotransmitter transport (Fig. 3c and Additional file 2: Table S7), suggesting that we are detecting true neuronal mC-expression associations despite measuring splicing in homogenate RNA-seq. Many of these genes were also differentially expressed between neuronal and glial nuclear RNA (FDR < 0.05, Table 1). Most, but not all, expression-associated cytosines at the gene- and exon level showed significant decreases in the expression as methylation levels increased.

The associations between these putatively regulatory cytosines and nearby expression levels can be explored in a web tool (https://jhubiostatistics.shinyapps.io/wgbsExprs/). The results can be interactively summarized such as in Table 1 for user-selected subsets and visualized as in Fig. 3b or via the UCSC genome browser (Additional file 1: Figure S14). By integrating neuronal mCpG and mCpH levels with accompanying RNA-seq data in the same brains, we have identified for the first time a direct association of hundreds of transcripts and their splicing events exclusively with mCpH, independent of mCpG levels, across the first two decades of human cortical development.

### DNAm patterns shed light on the active cell type and timing of neuropsychiatric phenotype development

**Fig. 4:**
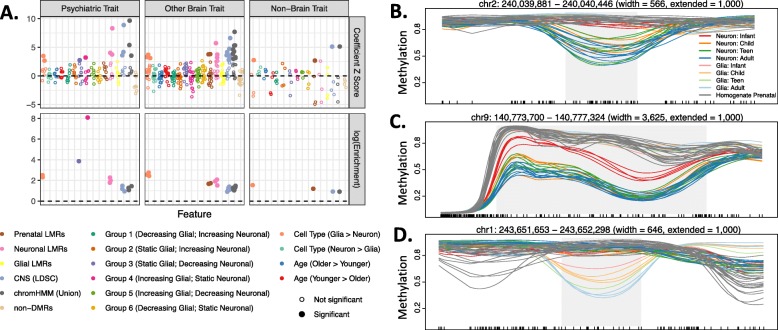
DNAm patterns and brain trait heritability. **a** Results assessing enrichment for heritability of 30 phenotypes within 16 groups of DNAm features using stratified linkage disequilibrium score regression (LDSC). Each dot represents the results for a single phenotype: DNAm feature pair. The color indicates the DNAm feature, and the phenotypes are stratified by column into psychiatric phenotypes, other brain-related phenotypes (i.e., neurological or behavioral-cognitive), or non-brain-related traits. The upper row shows the coefficient *z*-score for each tested phenotype: DNAm pair, or the amount of additional heritability explained by the DNAm feature over 53 baseline features in the model. The lower row shows the enrichment score or the proportion of heritability attributed to the feature divided by the proportion of SNPs in the feature. For clarity, enrichment scores of only the significant feature-trait combination are depicted. Filled in circles indicate significantly enriched heritability for a phenotype in a feature (coefficient *p* value corrected using Holms method ≤ 0.05). **b** A cdDMR overlapping *HDAC4*, a gene associated with autism spectrum disorder (ASD), shows the group 3 pattern of decreasing neuronal and static glial DNAm. **c** A cdDMR overlapping *CACNA1B*, a gene associated with ASD, shows the group 5 pattern of decreasing neuronal and increasing glial DNAm. **d** A cdDMR overlapping *AKT3*, a gene associated with schizophrenia, shows the group 6 pattern of decreasing glial and static neuronal DNAm. Gray shading indicates the boundaries of the cdDMR, and black tick marks on the *x*-axis indicate the position of CpGs

Previous work has attributed a high proportion of neuropsychiatric trait heritability to neuron-specific DNA methylation patterns [21]. Given the role of dynamic DNAm in marking DNA sequence function over the development, we examined the relationship between our methylation features and heritability for 30 human behavioral-cognitive traits, psychiatric and neurological disorders, and non-brain-related traits [22] (Additional file 2: Table S8), hypothesizing that DNAm patterns may illuminate not only the active cell type but potential critical time frames for genomic activity in these complex phenotypes. We used stratified linkage disequilibrium score regression (LDSC) [23] to estimate the proportion of heritability measured in GWAS summary statistics for each phenotype that could be attributed to each of 16 genomic features, including 10 sets of DMRs, LMRs identified in the prenatal, glial or neuronal methylome, human brain regulatory sequence annotated by chromHMM or the LDSC package, or non-differential CpG clusters (Fig. 4a). In agreement with previous findings [21, 24], human brain annotated regulatory sequence was broadly enriched for heritability of brain-specific traits (14 of 26 brain-associated phenotypes enriched in chromHMM or CNS (LDSC) regions at FDR ≤ 0.05), as were neuronal features (10 of 26 brain phenotypes enriched in neuronal hypomethylated regions, FDR ≤ 0.05; Additional file 2: Table S9). Significantly, differentially hypomethylated neuronal regions had on average 1.85 times higher enrichment scores than non-differential neuronal LMRs, meaning the DMRs explained 1.85× more heritability over regions containing a similar number of single nucleotide polymorphisms (SNPs) than the LMRs. Interestingly, body mass index (BMI) heritability was enriched in general brain regulatory sequence and hypomethylated neuronal DMRs (FDR ≤ 0.05), consistent with previous evidence linking this metabolic phenotype to regulatory sequence active in cells of the human central nervous system [23].

In terms of developmental DNAm patterns, heritability of BMI, IQ, neuroticism, and major depressive disorder was enriched in both postnatal neuronal and prenatal LMRs, suggesting early action of genetic influence on the development of these phenotypes (FDR ≤ 0.05). Few developmental differential groups captured a significant proportion of heritability for the 30 traits tested, perhaps because of their small size compared to the cell type-specific or non-differential groups (cdDMRs covered ~ 31, 240, and 838 times less sequence and included ~ 32, 273, and 904 times fewer SNPs than cell type DMRs, LMRs, or general brain features, respectively). However, despite covering only 607 kb, group 3 cdDMRs (i.e., static glial and decreasing neuronal DNAm) were significantly enriched for heritability of schizophrenia (coefficient *z*-score = 2.74, FDR = 0.039). Group 4 cdDMRs (111.7 kb; increasing glial, static neuronal DNAm) were also enriched for heritability of PTSD (coefficient *z*-score = 3.21, FDR = 0.01).

Given the enrichment for psychiatric disease heritability measured in common SNPs in these cdDMR groups, we then expanded our analysis to include seven curated gene sets containing de novo and rare inherited variation—including rare copy number variants (CNVs) and syndromic variants—associated with psychiatric, neurodevelopmental, and neurodegenerative disorders [25, 26]. We again found significant enrichment of hypomethylated neuronal DMRs in genes implicated in psychiatric and neurodevelopmental disorders (i.e., schizophrenia, autism spectrum disorder (ASD), syndromal neurodevelopmental disorders, and intellectual disability; all with OR > 2.04 and FDR < 1.9e−02; Additional file 2: Table S10). In this analysis, we also found an enrichment for hypermethylated neuronal DMRs in ASD genes from the SFARI Gene database and schizophrenia genes containing de novo mutations (both with OR > 1.92 and FDR < 5.0e−03). These results confirmed a prominent role of neuronal functioning in most of the neurodevelopmental disorders using an orthogonal measurement approach as done previously [24]. Over the postnatal development, group 5 cdDMRs (increasing glial, decreasing neuronal DNAm) were enriched in ASD genes from the SFARI Gene database (OR = 5.7, FDR = 4.1e−03), while group 6 cdDMRs (decreasing glial, static neuronal DNAm) were enriched in ASD, syndromal neurodevelopmental disorder, and intellectual disability genes (all with OR > 3.1 and FDR < 1.9e−02). In contrast, a curated set of neurodegenerative disorder genes showed no enrichment for cdDMRs, perhaps reflecting lesser relevance of the first two decades of postnatal epigenomic remodeling to the etiology of those disorders.

In the non-CpG context, we found significant enrichment of both increasing and decreasing mCpH levels in genes associated with schizophrenia, ASD, and syndromal neurodevelopmental disorders (all with OR > 2.1 and FDR < 2.0e−02). CpH hypomethylation in neurons was also enriched in the neurodegenerative disease gene set (OR = 2.6, FDR = 3.7e−03). Finally, significantly increasing mCpH was depleted in genes associated with intellectual disability (OR = 0.34, FDR = 3.7e−06). While enrichment for conflicting mCpH patterns is at first curious given the overall negative association between mCpH and gene expression, outside of the context of DMRs, individual mCpH could be associated both positively and negatively with expression. Indeed, many genes, exons, and PSI events whose expression both positively and negatively associated with both mCpH and mCpG were also enriched in genes associated with schizophrenia, ASD, and syndromal neurodevelopmental disorders (all with OR > 2.1 and FDR < 2.5e−02; Additional file 2: Table S11). Overall, these results suggest that these examples of dynamic methylation and associated isoform switching may play a role in the development of higher cognitive functions during brain maturation associated with these diseases.

## Discussion

Here, we have created a single-base resolution map of the dynamic DNAm landscape across the first two decades of postnatal human brain development in two cell type-enriched populations. Using FANS-derived samples, we were able to identify 40% more developmentally regulated regions of changing DNAm than were identified in homogenate DNAm cortical data. We profiled specific features of the DNAm landscape including LMRs, UMRs, PMDs, and DMVs and found that across features, neurons were typified by a general accumulation of mCpG. In the absence of complementary cell type-specific chromatin data, characterizing known DNAm features provided a more granular view of the potential functional genomic state in these regions than the available predictions derived from a few homogenate cortical samples. Particularly in studies using human postmortem brain, where tissue is often subjected to long postmortem intervals and low pH that degrades less stable epigenetic signatures, DNAm is a robust and durable marker that can be used to map the functional genomic terrain. These DNAm maps complement recently available epigenomic maps of different modalities generated on FANS-derived samples in the psychENCODE Consortium [27].

We further parsed the general accumulation of neuronal DNAm into six trajectories of cell type-specific developmental patterns and found that neuronal mCpG progressively diverged from a shared landscape with the glia and bulk prenatal cortex as the brain matured. Importantly, these diverging patterns were most striking during infancy through the first 5 years of postnatal life. The human brain experiences an explosion of synaptic connections during this time period, to nearly double the number found in the mature adult brain [28]. Although previous work has underscored this time frame in terms of rapid DNAm accumulation [1], this is the first work to refine DNAm patterns to reflect cell type-specific gain and loss of mCpG and mCpH within this critical window. By parsing these neuronal and glial DNAm patterns, we have highlighted epigenetically dynamic regions that may be contributing to the developmental processes such as synaptogenesis occurring during this time frame that establish the foundation for fine-tuning connections throughout the remainder of brain maturation. These results provide a finely resolved depiction of epigenetic plasticity being greatest during this period of life and support other evidence that environmental experience during these years may have an especially enduring impact on brain function [29].

mCpH is unusually abundant in neurons compared to other cell types and appears to undergo trinucleotide-specific reprogramming during differentiation from ESCs [17]. While most mCpH in ESCs occurs in the CAG context, neuronal mCpH predominantly accumulates in the CAC context [17]. Here, we elaborate on this relationship, showing that while both mCAG and mCAC aggregate in neurons as they mature and mCAG is gained faster than mCAC overall, mCAC accumulates proportionally faster in both neurons and glia over time. Interestingly, although neurons and glia contained mCpH in both trinucleotide contexts, mCAG was more likely to have higher levels in glia than neurons or be decreasing over the development; indeed, genes containing decreasing mCAG but not mCAC were strongly associated with neuronal biological processes. mCpH trinucleotide context, therefore, may have as yet not well-understood ramifications in brain development.

Because methylation levels in WGBS data are estimated by counting the number of cytosine (C) vs thymine (T) reads at any given cytosine site following bisulfite treatment, C/T SNPs at these sites may lead to the confounding of methylation results. However, only ~ 5% (*N* = 342,706) of the CpGs tested in this study that overlapped a SNP from dbSNP144 (~ 38.8% of 18.7 million CpGs overlapped a SNP with a minor allele frequency > 5%) were polymorphic in our samples, and the proportion was similar in the CpH context (~ 4% of CpH sites were polymorphic). Likewise, less than 1% of the CpGs significantly associated with cell type, age, or their interaction overlapped SNPs, a much smaller percent than the rest of the methylome. Therefore, we conclude that genetic variation at cytosines was not a significant confounder of methylation level assessment in our samples.

In terms of the relationship between mCpH and mCpG, we found that while neighboring mC (i.e., mCpG+mCpH) was not correlated genome-wide, mC was highly correlated within the cdDMRs despite local mCpH not being correlated. In other words, there was a convergence of levels of all contexts of methylation within the cdDMRs that was not detected genome-wide. mCpH also recapitulated the pattern seen in mCpG of diverging from a shared DNAm landscape with glia. Given that mCpH and mCpG have previously been shown to work in concert to recruit MECP2 binding to fine-tune gene expression [30], it is sensible that levels of both contexts would perhaps reflect a shared functional role within putatively regulatory cdDMR sequence, since cdDMRs were also enriched for gene bodies and brain enhancer sequence. This work quantifies this correlation for the first time, a DNAm relationship unique to only a selection of cell types including neurons.

The identification of widespread association of mCpG and mCpH with the expression and specific splicing events, particularly in neuronal genes enriched for neuropsychiatric diseases, highlights a potential novel role of mCpH and further expands the role of mCpG in the regulation of gene expression in neurons. Splicing is predominantly a co-transcriptional process influenced by changes in chromatin modifications and RNA-binding proteins; the effects of DNAm on splicing decisions are not yet well studied [31]. Here, we found thousands of associations between mC levels and gene, exon, and PSI expression in developing postnatal neurons, particularly featuring many exons that are exclusively associated with mCpH. Although it is not possible to establish a causal role for mC in these data, these analyses, which are summarized in the provided website, can empower other researchers to explore the connection between DNAm and alternative isoform use, a phenomenon particularly prevalent in the developing brain that is often associated with disease [20].

We also explored the relationship between DNAm patterns and genetic associations with various phenotypes and found both expected and surprising associations. We confirmed enrichment for heritability of brain traits generally in neurons and heritability for schizophrenia, a disorder with strong neurodevelopmental underpinnings, specifically in genomic regions losing DNAm preferentially in neurons over early postnatal development (i.e., group 3 cdDMRs). This result emphasizes the critical nature of neuronal development and maturation in the early establishment of pathological connectivity and function for this adult-onset disorder, as most DNAm loss—generally associated with increased activity of a gene or regulatory element—occurred within the first five postnatal years. Indeed, group 3 cdDMRs were present in genes such as *GRIN1*, *SYN1*, and *CAMK2A*, and others involved in establishing synapse organization and function, a hallmark of early postnatal brain development, implicating abnormal genetic regulation of neuronal connectivity in schizophrenia development. Interestingly, heritability for PTSD was significantly enriched in regions preferentially gaining DNAm in the non-neurons over the development (group 4 cdDMRs), regions associated with neural precursor cell proliferation, and cell-cell connective properties (Fig. 1c). This association, particularly given the small amount of sequence covered by group 4 cdDMRs, could lead to fruitful insights into susceptibility for PTSD and warrants further study.

We also found enrichment of genes associated with rare variants implicated in psychiatric and neurodevelopmental disease in a variety of cell types and developmental trajectories, highlighting the genomic boundaries, developmental timing, and cellular context of epigenomic remodeling of regulatory elements or expressed features associated with known risk genes. Two examples of this are *HDAC4* and *CACNA1B*, genes associated with ASD in the SFARI Gene database. We identified a 566-bp group 3 (decreasing neuronal, static glial DNAm) cdDMR within an intron of *HDAC4*, a calcium-sensitive transcriptional repressor, and a 3.6-kb group 5 (decreasing neuronal, increasing glial DNAm) cdDMR within *CACNA1B*, a gene encoding a voltage-gated calcium channel subunit (Fig. 4b, c). Even though both genes are implicated in ASD and both cdDMRs are hypomethylated in neurons, the timing of loss of mCpG suggests that *CACNA1B* activity occurs earlier in postnatal development than *HDAC4*. Given that ASD onset is typically in early childhood, these risk genes may therefore have differing implications in the etiology of ASD. Another example is a 646-bp group 6 (decreasing glial, static neuronal DNAm) cdDMR that overlaps the last intron and exon of *AKT3*, a serine/threonine-protein kinase gene. Although the *AKT3*/1q44 locus has been associated with schizophrenia risk, the mechanisms are not yet known given that *AKT3* is involved in many biological functions [32]. Interestingly, this cdDMR selectively lost mCpG in the glial samples beginning in infancy, suggesting that *AKT3* activity in human DLPFC may be localized to glia beginning in infancy or earlier (Fig. 4d). This work provides the first ex vivo look at DNAm dynamics within human neurons and glia and thus allows for the first examination of these parameters within the relevant organ, the brain.

Despite these insights, our data invoke several caveats. While NeuN-based FANS greatly improves identifying developmental DNAm changes over homogenate data, designating NeuN+ and NeuN− samples as “neurons” and “glia,” respectively, is not completely accurate in that NeuN− samples will include the signal from unlabeled neurons and mask non-neuronal diversity. However, recent work assessing the brain regional DNAm differences between NeuN+ and NeuN− found that NeuN− contributed comparatively marginal variability in DNAm compared to NeuN+, suggesting that neuronal methylomes are much more dynamic than non-neurons [21]. Likewise, while a percentage of the bases in the DMRs identified in this work has also been previously shown to be differentially methylated by neuronal subtypes whose unique methylomes are masked using NeuN-based FANS [5, 14, 33], the proportion of these subtypes should be stable over postnatal development [34]. Future epigenomics studies however can improve on the resolution of our study by isolating more specific neuronal subpopulations to refine the cellular specificity of these neuronal methylation changes largely occurring in the first few years of life.

Another caveat is that WGBS does not allow for the discrimination between mC and hydroxymethyl-cytosines (hmC), an intermediary in the demethylation pathway. Previous work has shown [5] that only a fraction of CpGs have measurable levels of hmC, suggesting that our results are not confounded. In the cited study [5], hmCpG signal from the homogenate cortex represented 10% of the hypermethylation found in excitatory neuron mCpG, suggesting that most of the mCpG signal in our neuronal data likely is true mCpG. The level of hmC in the non-CpG context remains controversial, with some studies not identifying hmCpH [5] and others detecting low amounts (1% in FANS-derived human glutamatergic and 0.47% in GABAergic neurons) [35]. Further, FANS-derived human oligodendrocytes showed little hmC in the same study [35]. Overlap of our cdDMRs with DMRs between neurons and oligodendrocytes in a study of mC in FANS-derived human PFC samples [35] showed that cell type differences primarily reflected true mC rather than hmC contamination in WGBS, while hmC DMRs between neuronal subtypes primarily were reflected in hypomethylated neuronal cdDMR groups and not the hypermethylated neuronal groups that would potentially include hmC signal contamination (Additional file 1: Figure S7B). Future work, however, should more closely examine the contribution of hmC to the global hypermethylation seen during neurodevelopment.

## Conclusions

By mapping the changing DNAm landscape over human postnatal neuronal and glial development, we have identified unique trajectories of DNAm change particularly dynamic during the first 5 years of life which show convergence between mCpG and mCpH, as well as associations between single mCpG and mCpH and alternative splicing. These patterns may also help illuminate the mechanisms through which psychological, neurological, and psychiatric traits are developed by placing known genetic contribution in an epigenomic context.

## Methods

### Postmortem brain tissue acquisition and processing

Homogenate postmortem tissue of the prefrontal cortex (dorsolateral prefrontal cortex, DLPFC, BA46/9) was collected from 24 postnatal and 20 prenatal donors. Clinical characterization, diagnosis, and macro- and microscopic neuropathological examinations were performed on all samples using a standardized paradigm, and subjects with evidence of macro- or microscopic neuropathology were excluded, as were all subjects with any psychiatric diagnoses. Details of tissue acquisition, handling, processing, dissection, clinical characterization, diagnosis, neuropathological examinations, and quality control measures have been further described previously [36].

### Fluorescence-activated nuclei sorting

The nuclei were isolated from 100 to 300 mg of pulverized DLPFC tissue using dounce homogenization followed by ultracentrifugation over a sucrose density gradient. Homogenization was performed on ice in 5 mL lysis buffer [0.32 M sucrose, 3 mM magnesium acetate, 5 mM calcium chloride, 5 mM EDTA (pH 8.0), 10 mM Tris-HCl (pH 8.0), 0.1% Triton X-100], and the resulting homogenate was layered over 38 mL sucrose buffer [1.8 M sucrose, 3 mM magnesium acetate, 10 mM Tris-HCl (pH 8.0)] and centrifuged at 139,800×*g* for 2 h at 4 °C. Cellular debris and lysis and sucrose buffers were removed, and the pelleted nuclei were resuspended in 500 μL PBS. the nuclei were then labeled in a solution of anti-NeuN antibody conjugated to Alexa Fluor 488 (A60, Millipore, 1/1000) and 0.1% BSA, rocking for 30 min at 4 °C, followed by the addition of DAPI. Nuclei sorting was performed at the Johns Hopkins School of Public Health Flow Cytometry Core with a MoFlo Legacy (Beckman Coulter) using Summit (version 4.3) software. The purity of the sorted populations was determined to be > 99% based on resorting NeuN+ and NeuN− populations through the same gates.

The identity of the NeuN+ and NeuN− populations as neuron-enriched and glia-enriched, respectively, was confirmed by sequencing nuclear RNA from each population and determining that neuronal and glial biological processes were enriched in genes differentially expressed between the two groups (FDR < 0.05; Additional file 1: Figure S1B). Likewise, cell type marker gene expression patterns also corroborated the neuronal- and glial-enriched identities of NeuN+ and NeuN− samples, respectively (Additional file 1: Figure S1C). The estimated proportion of neurons in homogenate DNA methylation data based on deconvolution using differentially methylated sites between NeuN+ and NeuN− samples was highly correlated with the empirical proportion of neurons (Additional file 1: Figure S1D). Raw sorting data is shown in Additional file 1: Figure S15.

### Whole genome bisulfite library preparation and sequencing

Genomic DNA extraction was performed using the DNeasy Blood and Tissue Kit (Qiagen). Bisulfite conversion of 600 ng genomic DNA was performed with the EZ DNA methylation kit (Zymo Research). Sequencing libraries were made with Illumina TruSeq DNA Methylation library preparation kits. Lambda DNA sequence was spiked in at 1% concentration to assess the bisulfite conversion efficiency. Library concentrations were measured using a NanoDrop, and library fragment sizes were measured using an Agilent Bioanalyzer 2100. Libraries were spiked with 10% PhiX to improve base calibration calls and subsequently sequenced on an Illumina X-Ten Platform with paired-end (PE) reads (2 × 150 bp), targeting 30× coverage and Q30 > 70% read quality.

### RNA-seq library preparation and sequencing

RNA was extracted from homogenate and sorted samples using TRIzol LS Reagent (Thermo Fisher Scientific) followed by the RNeasy MinElute Cleanup Kit (Qiagen). RNA sequencing libraries were made with the TruSeq RNA Library Prep Kit (Illumina) and the RiboGone Low-Input Ribosomal RNA Removal Kit (Clontech). Library concentrations were measured using a Qubit 2.0, and library fragment sizes were measured using Caliper Life Sciences LabChip GX. One hundred-base-pair PE sequencing was run on an Illumina HiSeq 2000.

### WGBS data processing/alignment

We aligned the PE reads for each sample to the in silico bisulfite-treated hg19 genome, which we created using the Bismark v0.15.0 [37] bismark_genome_preparation program. For each library of PE reads (one per sample), the following processing was performed (Additional file 1: Figure S16):

- FastQC v0.11.4, to assess the read quality, presence of adapter sequence, and overrepresented sequences.
- Trimmomatic v0.35 [38], to trim low-quality and adapter-containing portions of the reads, with the following parameters: PE -threads 12 -phred33 ILLUMINACLIP:/Trimmomatic-0.35/adapters/TruSeq3-PE.fa:2:30:10:1 LEADING:3 TRAILING:3 SLIDINGWINDOW:4:15 MINLEN:75. This resulted in three sub-libraries of reads per sample: one PE sub-library, and two single-end sub-libraries where the corresponding paired read was trimmed to a length below the defined threshold.
- FastQC v0.11.4, on each of the three sub-libraries, to assess the improvement in read quality and adapter content following trimming.
- FLASh v1.2.11 [39], to merge the PE sub-library reads into longer single-end reads, as reads that overlapped around CpGs and CpHs might bias or at least double-count the DNAm estimates. Furthermore, Bismark [37] could only be run on single- or paired-end reads and not a combination of both. This further split the PE sub-library into three sub-libraries: the subset of PE reads that were merged into longer single-end reads and then left and right single-end reads that could not be merged.
- Bismark v0.15.0 [37], to align each of the five now-single-end sub-libraries (left-trimmed, right-trimmed, FLASh-merged, FLASh-left-unmerged, and FLASh-right-unmerged) to the bisulfite-converted hg19 genome using bowtie2 [40] and the --non-directional argument.
- Resulting alignment (BAM) files across five sub-libraries were merged, sorted, and indexed using samtools v1.3 [41] to produce one large/merged BAM file per sample.
- Alignments with evidence of duplication were removed using the MarkDuplicates program in Picard tools v1.141, which systematically appeared to be localized to low complexity DNA sequence near centromeres.
- The Bismark [37] bismark_methylation_extractor program was run on each post-duplicate-removed BAM file per sample to extract CpG and CpH DNAm levels.

We additionally aligned reads from each sample to the PhiX and Lambda genomes to compute quality control metrics related to sequencing and bisulfite conversion quality. The average percentage of reads mapping back to the Lambda genome was 1.32%, and the average bisulfite conversion efficiency was 98.64%. The average bisulfite conversion efficiency was not associated (*p* > 0.05) with cell type, age, cell type (adjusting for age), age (adjusting for cell type), and the interaction of them in the NeuN− (glia) and NeuN+ (neuron) samples as well as for age in the homogenate samples. The genome coverage decreased from an initial average of 43× to 10× across the processing stages as shown in Additional file 1: Figure S17 (coverage). For each of the processing stages, there was no significant difference between cell types (adjusting for age), age (adjusting for cell type), and the interaction between age and cell type (*p* Bonferroni > 0.05). The genome coverage was extracted from the FASTQC reports and by using bamcount v0.2.6. [42]

We processed the prenatal and postnatal homogenate brain samples using the same procedure described above to produce Bismark [37] report files. Then, using bsseq [43], we extracted the methylation values for the CpG positions observed in our postnatal sorted samples in order to make them comparable to each other by filtering to CpGs with coverage in all 55 postnatal samples.

### RNA-seq data processing

Raw sequencing reads were mapped to the hg19/GRCh37 human reference genome with splice-aware aligner HISAT2 v2.0.4 [44]. Feature-level quantification based on GENCODE release 25 (GRCh38.p7) annotation on hg19 coordinates was run on aligned reads using featureCounts (subread v1.5.0-p3) [45]. Using custom R code, we processed the different feature counts and created RangedSummarizedExperiment objects using the SummarizedExperiment Bioconductor package v1.4.0. We calculated the “percent spliced in” (PSI) using the SGSeq [46] Bioconductor package v1.12.0 and the Gencode v25 annotation for the GRCh37 human reference genome (*ftp://**ftp.ebi.ac.uk/pub/databases/gencode/Gencode_human/release_25/GRCh37_mapping/gencode.v25lift37.annotation.gtf.gz*) from the BAM files generated by HISAT2. We used default arguments except for the function analyzeVariants() where we used a min_denominator=10.

### Comparing homogenate vs cell type-specific WGBS

We first filtered CpGs to those with coverage in all 55 postnatal samples. For homogenate samples, we used lmFit() and ebayes() from the limma [47] Bioconductor package v3.34.5 to assess the age-associated changes to DNAm levels with the linear model ~ Age. For the cell type-specific samples, we used a linear model ~ Age * Cell Type to assess cell type, overall age, and age in a cell type changes to DNAm levels. We subset CpGs to those that were significantly differentially methylated by age in homogenate samples (*p* < 1 × 10^−4^) and plotted the coefficients for each CpG in Additional file 1: Figure S2A-D. The relationship between each variable was quantified using Fisher’s exact test.

### Identifying methylation features

We identified PMDs, UMRs, and LMRs using the bioconductor package MethylSeekR (version 1.20.0). We obtained coverage information from the cleaned set of ~ 18 million CpGs by extracting coverage and methylation using the getCoverage() function from the bsseq bioconductor package [43] v1.10.0. PMDs were called using segmentPMDs() and were visually inspected using plotPMDSegmentation(). To create a more stringent cutoff for PMDs, we filtered PMDs to those longer than 100 Kbp. We calculated the FDRs using calculateFDRs(), while masking the > 100 Kbp PMDs, setting the *m* parameter to 0.5 and the FDR cutoff to 10. PMDs were further filtered to exclude overlaps with the UCSC “gap” database table from hg19 except for the gaps labeled as heterochromatin. We calculated UMRs and LMRs using segmentUMRsLMRs(). DMVs were defined as UMRs in which pmeth was less than or equal to 0.15, and the width was greater than or equal to 5 Kbp.

### Identifying CpG differentially methylated regions

Using the bsseq [43] Bioconductor package v1.10.0, we loaded the Bismark [37] report files and filtered the CpG data to keep only the bases where all samples had a minimum coverage of 3 (18,664,892 number passed the filter). We smoothed the methylation values of the remaining CpGs using the BSmooth() function from bsseq with the parallelBy = “sample” option. To identify the age and cell type DMRs, we used a model that adjusted for both covariates while for the interaction model, we included an additional interaction covariate. We identified the DMRs using the bumphunter [48] Bioconductor package v1.14.0 using the maxGap = 1000, B = 250, nullMethod = “permutation,” smooth = FALSE options, which tends to be conservative in DMR identification [48]. For the cutoff option, we used 0.1 for the cell type DMRs adjusting for age, 0.005 for the age DMRs adjusting for cell type, and 0.009 for the age and cell type interaction DMRs. These first two parameter cutoffs correspond to 10% minimum DNAm differences between neurons and glia and 5% change in DNAm per decade of life across cell types and were chosen based on functionally relevant change in DNAm. The cutoff for the interaction model (cdDMRs) was based on selecting the equivalent percentile of change from the overall age model (86th percentile)—this percentile-based cutoff was in line with recommendations for selecting cutoffs for statistical models with less clear biological interpretations [48]. We used a family wise error rate (FWER) threshold of 5% to determine the DMRs: fwer output from the bumphunter() function. A small subset of DMRs involved a single CpG, which arises from having a more significant area (length times effect size) than any DMRs identified in null permuted data. All DMRs showed less than 10% median percent absolute bias to the technical and biological covariates as shown in Additional file 1: Figure S18 (sensitivity).

### cdDMR processing

For the “interaction” DMRs with FWER< 5% (i.e., cdDMRs), we extracted the methylation values from the glial and neuronal samples using bsseq [43] v1.13.9 and then computed a mean methylation value per DMR for each cell type. We also calculated the mean interaction coefficient for each DMR across all the cytosines in the DMR by cell type. Using the mean coefficients by cell type, we clustered the interaction DMRs using the kmeans() function with centers = 6 and nstart = 100 options. We chose centers = 6 based on the biological interpretability of the results and because *k* = 6 results in an optimal AIC for clusters computed with mean centered and scaled data. For each cytosine in the cdDMRs, we calculated the *t*-statistic and coefficient for age explaining the differences in methylation adjusted for cell type: ~ age + cell type. For each DMR, we computed the mean age coefficient by cell type and then calculated the median absolute coefficient across all cdDMRs. The neuronal/glia ratio is 1.5 for such median absolute age effects across the cdDMRs.

Plots for the DMRs were made using bsseq [43] v1.14.0, EnsDb.Hsapiens.v75 v2.99.0, and RColorBrewer v1.1-2. Genes within 20 kb of a DMR were retained for the plots. Genes and exons were included using the annoTrack argument, and we used extend = 2000 for making the plots.

### Roadmap epigenome enrichments

We computed the relative enrichments of different genomic regions using Epigenome Roadmap data [12] by computing the proportion of bases in each of the 15 ChromHMM states for each of the cells and tissues provided by the Consortium. We compared the proportion of bases in each state within each candidate region set to the overall genome and computed the corresponding log2 enrichments between the regions and this genomic background. We compared DMR and mCpG-based methylation feature regions to all profiled cell types in the Consortium for these analyses.

### Assessing the contribution of neuronal subtypes

The percent of neuronal subtype-specific bases was calculated by reducing the total subtype-specific CpG-DMRs from Luo et al. [14], reducing the bases in each group of cdDMRs and calculating the percent of cdDMR bases that intersected the merged subtype-specific CpG-DMR bases.

### Enrichments for HARs and enhancers

We calculated the enrichment of genomic segments overlapping cdDMRs and human accelerated regions (HARs) and enhancers [15] using Fisher’s exact test. We calculated the overlap of methylation features (DMRs, mCpH, expression-associated cytosines) and HARs or enhancers with the entire set of CpG clusters used to identify DMRs as background. We corrected for multiple testing using the false discovery rate (FDR).

### Gene Ontology analyses

Gene Ontology enrichment analyses were performed using clusterProfiler [49] v3.6.0 using the options pAdjustMethod = “BH,” pvalueCutoff = 0.1, and qvalueCutoff = 0.05 on the Entrez IDs for each expression feature to test for enriched biological processes (BP), cellular compartments (CC), and molecular functions (MF). Only cytosines or DMRs overlapping genes were included.

### CpH processing

Using Bismark v0.16.3 [37], we created report files using the methylation extractor program with the CX_context and split_by_chromosome options for the hg19 human genome in order to extract the methylation values for the CpHs. Then, for each chromosome, using the bsseq [43] Bioconductor package v1.10.0, we loaded the Bismark [37] report files and added the c_context and trinucleotide_context information from Bismark using custom R code based on the bsseq internal code that uses the data.table package v1.10.4. After combining the results for each chromosome, we filtered the CpHs to keep only those where all samples had a minimum coverage of 5 (58,109,566 number passed the filter).

We chose the coverage thresholds of 5 reads for CpHs rather than the 3 used for CpGs earlier to be more conservative in the CpHs that would be included, given the number of tests that were performed in that context. Despite the difference in the cutoffs, however, mean coverage levels across the cytosines meeting these thresholds were similar between CpGs and CpHs (median CpG coverage = 13, IQR 10.6–15.8; median CpH coverage = 11.7, IQR 10.3–13.3).

### Global autocorrelation

Using CpG and CpH positions with a minimum coverage of 3 and 5, respectively, for all samples, we calculated the autocorrelation for the methylation levels for the CpGs, the CpHs, the CpHs with a CHG trinucleotide context, or the CpHs with a CHH trinucleotide context. For each of the sets, we grouped the positions using derfinder v1.12.0 into groups by a maximum distance of 1 kb. Only those groups with at least 5 Cs were further considered. For each sample, we then calculated the autocorrelation using the acf() function with lag.max = 4 in parallel for each chromosome using BiocParallel v1.12.0. For each cluster of cytosines, we calculated the mean across the neuronal (NeuN+) and the glial (NeuN−) samples at each autocorrelation lag. After combining and tidying the results, we visualized the global auto correlation using ggplot2 v2.2.1. We repeated this same analysis for the Lister et al. data [1].

### Autocorrelation within DMRs

Similar to the global autocorrelation, we extracted the methylation values at CpGs with a minimum coverage of 3 and the CpHs with a minimum coverage of 5 that were within each of the sets of DMRs (age, cell type, or interaction). We then computed the autocorrelation for DMRs with a least 5 different cytosines using the acf() function with a lag.max = 4 and calculated the mean auto-correlation among the neuronal and glial samples. The lag is proportional to the genomic distance as shown in Additional file 1: Figure S19 (lag and distance).

### Lister et al. [1] data processing

We downloaded the WGBS data from Lister et al. [1] (SRA accession SRP026048). We then processed and aligned the data following the same steps we used for our data. Using bsseq [43] as in the “CpH processing” section, we extracted the methylation values from the Bismark [37] report files and added the c_context and trinucleotide_context information per chromosome. We then merged the results for all the chromosomes retaining only the CpG and CpH positions we observed with a minimum coverage of 3 and 5 in our data, respectively. To assess the replication of our cell type DMR results, we computed the mean methylation differences across the CpG positions comparing neuron and non-neuron samples in the Lister et al. data [1] using the rowttests() function from the genefilter package version 1.56.0. We then computed the mean difference for each of the DMRs and compared this mean difference against the DMR mean methylation difference derived from our data to derive the concordance and correlation between them. To assess the replication of our age DMR results, we modeled age as a continuous variable and calculated the mean methylation difference per year for every CpG contained in the age DMRs using lmFit() function from limma. Finally, we compared the mean methylation difference in the Lister et al. data [1] against the observed mean methylation difference for the age DMRs we derived.

### Identification of differentially methylated positions

With the set of CpGs and CpHs with a minimum coverage 3 and 5 in all samples, respectively, and the same models for identifying the DMRs, we identified the differentially methylated positions (DMPs), keeping the CpGs and CpHs separate. For the CpHs, we further filtered to keep only those where at least 5 samples had a methylation value greater than 0 (40,818,742 or 70.2%). We used the limma Bioconductor package v3.30.13 for determining the DMPs by running the functions lmFit() and eBayes() with default parameters and FDR < 5%.

### Differential expression between cell types

We combined the gene counts for the polyA+ and RiboZero sequencing protocols for the sorted RNA-seq data: 3 NeuN+ and 3 NeuN− samples for a total of 12 RNA-seq sequencing runs. We calculated the library size normalization factors using calcNormFactors() from edgeR [50] (v3.22.3) and identified differentially expressed genes using voom(), lmFit(), and eBayes() from limma [47, 51]. We repeated this procedure for the exon counts. We chose cell type-specific marker genes to check for the correct expression patterns from a database for cell type-specific RNA-seq identified in mouse [52].

### Methylation vs expression associations

With the sorted RNA-seq data, we computed the average gene expression per age group (infant, child, teen) and cell type (six groups) and correlated these values to average DNAm levels in the same groups in both the CpG and CpH contexts at the gene promoter and body, exon (500 bp window), and splice junction (50 bp into each intron) levels. With the RangedSummarizedExperiment objects with the RNA-seq polyA homogenate data and the bsseq objects with the CpG and CpH data, we determined which CpG and CpH positions explained the changes in expression (FPKM) at the gene or exon level as well as in percent spliced in (PSI). We retained only the expression and PSI data from the postnatal samples and matched them by brain identifier to the neuronal methylation data with a final sample size of 22. We filtered lowly expressed features using the expression_cutoff() function from the jaffelab package v0.99.18: mean FPKM > 0.22 mean for genes and 0.26 for exons. For genes and exons, we transformed the expression values to log2(FPKM + 1) and extracted the raw PSI values. Using MatrixEQTL [53] v2.2 (GitHub b9a9f01 patch), we then identified the methylation quantitative trait loci (QTL) for the CpG and CpH methylation data separately using the function Matrix_eQTL_main()function with options pvOutputThreshold = 0, pvOutputThreshold.cis = 5e−4, useModel = modelLINEAR, and cisDist = 1000. We identified marginal CpG associations near CpH associations by running MatrixEQTL again for the CpG in a 1 kb window around the CpH positions with an association with expression at FDR < 5% for each expression feature type using the same parameters as above except for pvOutputThreshold.cis = 0.01. We filtered the associations to retain only those having at least 11 samples with non-zero methylation and 11 samples with non-one methylation values to remove extreme cases. We further restricted the results to protein-coding genes and dropped any with infinite *t*-statistics. To assess whether age confounds the relationship between methylation and expression, we used a multiple linear regression model adjusting for age and checked if the methylation coefficient was still FDR < 5%. Venn diagrams in Fig. 3 were made with the VennDiagram package v1.6.18. For the gene and PSI associations, we used the gene ID to check if it was present in the 3473 differentially expressed genes from the sorted RNA-seq data (described above) with higher expression in neurons and the top 5000 DE genes with higher expression in glia at FDR < 5%; similarly, we did so for exons and the top 5000 DE exons (FDR < 5%) with higher expression in each cell type. The LIBD WBGS Expression explorer at https://jhubiostatistics.shinyapps.io/wgbsExprs/ was made using the bsseq [43] v1.14.0, DT v0.4, SGSeq [46] v1.12.0 and shiny v1.0.5 R packages.

### Stratified linkage disequilibrium score regression

GWAS summary statistics for 30 phenotypes [21, 22] were downloaded from the sources listed in Additional file 2: Table S8. We used LDSC (LD SCore) v1.0.0 to estimate the proportion of heritability captured in 16 sets of genomic DNAm features for each GWAS phenotype, including the DMRs and LMRs defined above in this work, non-differentially methylated CpG clusters (called above before the DMR analysis, excluding DMR sequence), central nervous system annotations included in the LDSC package (referred to as CNS (LDSC)), and regions annotated as putatively regulatory in the human brain using chromHMM (i.e., the union of regions annotated as “Bivalent Enhancer,” “Bivalent/Poised TSS,” “Genic enhancers,” “Flanking Active TSS,” “Active TSS,” “Strong transcription,” and “Enhancers” in the following tracks accessed using the AnnotationHub Bioconductor package (v2.14.5) [54]: AH46920, AH46921, AH46922, AH46923, AH46924, AH46925, AH46926, AH46927, AH46934, and AH46935).

We first converted the GWAS summary statistics into the .sumstats format using munge_sumstats.py, keeping only HapMap 3 SNPs (downloaded from https://data.broadinstitute.org/alkesgroup/LDSCORE/w_hm3.snplist.bz2) as described in the Partitioned Heritability LDSC tutorial. We made .annot files for each custom feature set based on the list of SNPs in the CNS cell type annotations provided in the LDSC package and estimated partitioned LD scores for each feature using 1000 Genomes plink files (downloaded from https://data.broadinstitute.org/alkesgroup/LDSCORE/1000G_Phase3_plinkfiles.tgz) using ldsc.py. We finally estimated the partitioned heritability for each feature-phenotype combination by adding each feature individually to the “baseline model” including 53 baseline annotations described in Finucane et al. [23].

### Enrichments for genes associated with brain disorders

We calculated the enrichment of genes overlapping different methylation features in gene sets described by Birnbaum et al. [25]. We measured what fraction of the genes in each set overlapped methylation features (i.e., DMRs, mCpH, and expression-associated cytosines) with Fisher’s exact test using all expressed genes with Entrez IDs as background. We corrected for testing multiple disorder gene sets using the false discovery rate (FDR).

## Additional files

Additional file 1: **Figure S1**. Confirmation of neuronal- and glial-enriched identity of NeuN+ and NeuN- samples. **Figure S2**. Detecting developmental changes in homogenate vs. cell type-specific DNAm data. **Figure S3**. Unmethylated Regions (UMRs) and Low-methylated regions (LMRs). **Figure S4**. Partially methylated domains (PMDs). **Figure S5**. DNA methylation valleys (DMVs). **Figure S6**. Differentially methylated regions (DMRs). **Figure S7**. Cell type-specific developmental DMRs and neuronal subtype methylation and hydroxymethylation. **Figure S8**. cdDMR overlap of human brain developmental enhancers and Human Accelerated Regions (HARs). **Figure S9**. Cell type-specific, developmentally dynamic DMRs (cdDMRs) and epigenetic states. **Figure S10**. CpH methylation distribution, levels and context-specific biological process ontology. **Figure S11**. Trajectories of CpH methylation accumulation in cdDMR groups. **Figure S12**. Relationship between methylation and expression. **Figure S13**. Cellular compartment ontology. **Figure S14**. Web meQTL browser display. **Figure S15**. Raw sort data. **Figure S16**. Data processing/alignment pipeline. **Figure S17**. Genome coverage across processing stages. **Figure S18**. DMR sensitivity analyses. **Figure S19**. genome distance versus autocorrelation lag. (PDF 18992 kb)

Additional file 2: **Table S1**. WGBS Phenotype, Sequencing Data. **Table S2**. RNA Phenotype, Sequencing Data. **Table S3**. Number of cytosines measured and distribution of methylation by context. **Table S4**. Number of Low Methylation Regions (LMRs) and Unmethylated Regions (UMRs) per sample. **Table S5**. Cell type-specific, developmental differentially methylated regions (cdDMRs). **Table S6**. mC association with cell type and age in postnatal cell type-specific samples. **Table S7**. Molecular function gene ontology enrichment for genes including exons whose expression is associated with cytosine methylation levels. **Table S8**. GWAS traits assessed using LDSC. **Table S9**. Stratified linkage disequilibrium score regression results. **Table S10**. Enrichment of DMRs and mCpH for disease-associated gene sets. **Table S11**. Enrichment for disease gene sets in DNAm-splicing association features. **Table S12**. Variable dictionary for Table 1. (XLSX 437 kb)

Additional file 3: Review history. (DOCX 22 kb)

## Data Availability

Raw and processed nucleic acid sequencing data generated to support the findings of this study are part of the PsychENCODE Consortium and the Brainseq Consortium data releases. Specifically, WGBS data have been deposited at www.Synapse.org along with the other PsychENCODE data, under the accession code syn5842535 [55]. The homogenate RNA-seq samples were also part of a larger study of RNA-seq data from homogenate DLPFC tissue (BrainSeq Consortium Phase I), which was also deposited at www.synapse.org and summarized in http://eqtl.brainseq.org/phase1 [56]. The processed, homogenate RNA-seq data for this study have additionally been deposited via Globus under the jhpce#brainepi-cellsorted collection at the following location: http://research.libd.org/globus/jhpce_brainepi-cellsorted/. NeuN-sorted RNA-seq data were originally published as part of phase II of the Brainseq Consortium (http://eqtl.brainseq.org/phase2/) and have also been deposited via Globus under the jhpce#brainepi-polyA collection at the following location: http://research.libd.org/globus/jhpce_brainepi-polyA/. Publicly available data reprocessed in support of the conclusions in this work were downloaded from the Gene Expression Omnibus under GEO accession GSE47966 [1]. Code used in the analyses included in this paper is available through GitHub (https://github.com/LieberInstitute/brain-epigenomics), and the version of the source code used in this manuscript is deposited in Zenodo (10.5281/zenodo.3375593) [57].
