# Supplementary material for: Divergent neuronal DNA methylation patterns across human cortical development reveal critical periods and a unique role of CpH methylation
Source: Genome Biol. 2019 Sep 26;20:196. doi: 10.1186/s13059-019-1805-1 (PMC6761727; doi:10.1186/s13059-019-1805-1)
Supplement: Supplementary file 1 — Figure S1. Confirmation of neuronal- and glial-enriched identity of NeuN+ and NeuN- samples. Figure S2. Detecting developmental changes in homogenate vs. cell type-specific DNAm data. Figure S3. Unmethylated Regions (UMRs) and Low-methylated regions (LMRs). Figure S4. Partially methylated domains (PMDs). Figure S5. DNA methylation valleys (DMVs). Figure S6. Differentially methylated regions (DMRs). Figure S7. Cell type-specific developmental DMRs and neuronal subtype methylation and hydroxymethylation. Figure S8. cdDMR overlap of human brain developmental enhancers and Human Accelerated Regions (HARs). Figure S9. Cell type-specific, developmentally dynamic DMRs (cdDMRs) and epigenetic states. Figure S10. CpH methylation distribution, levels and context-specific biological process ontology. Figure S11. Trajectories of CpH methylation accumulation in cdDMR groups. Figure S12. Relationship between methylation and expression. Figure S13. Cellular compartment ontology. Figure S14. Web meQTL browser display. Figure S15. Raw sort data. Figure S16. Data processing/alignment pipeline. Figure S17. Genome coverage across processing stages. Figure S18. DMR sensitivity analyses. Figure S19. genome distance versus autocorrelation lag. (PDF 18992 kb) [file 13059_2019_1805_MOESM1_ESM.pdf]

Supplemental Figures

Title: **Divergent neuronal DNA methylation patterns across human cortical development reveal critical periods and a unique role of CpH methylation**

Authors: Price AJ[1,2]*, Collado-Torres L[1,3]*, Ivanov NA[1], Xia W[1], Burke EE[1], Shin JH[1], Tao R[1], Ma L[1], Jia Y[1], Hyde TM[1,4,5], Kleinman JE[1,5], Weinberger DR[1,2,4,5,6], Jaffe AE[1,2,3,7,8,+]

Affiliations:

1. Lieber Institute for Brain Development, Johns Hopkins Medical Campus, Baltimore, MD, USA
2. McKusick-Nathans Institute of Genetic Medicine, Johns Hopkins University School of Medicine (JHSOM), Baltimore, MD, USA
3. Center for Computational Biology, Johns Hopkins University, Baltimore, MD, USA
4. Department of Neurology, JHSOM, Baltimore, MD, USA
5. Department of Psychiatry, JHSOM, Baltimore, MD, USA
6. Department of Neuroscience, JHSOM, Baltimore, MD, USA
7. Department of Mental Health, Johns Hopkins Bloomberg School of Public Health (JHSPH), Baltimore, MD, USA
8. Department of Biostatistics, JHSPH, Baltimore, MD, USA

*equally contributing authors

Corresponding Author: Andrew E Jaffe, 855 N Wolfe St, Ste 300; Baltimore MD 21205. Phone: 1-443-287-6864; Email: andrew.jaffe@libd.org

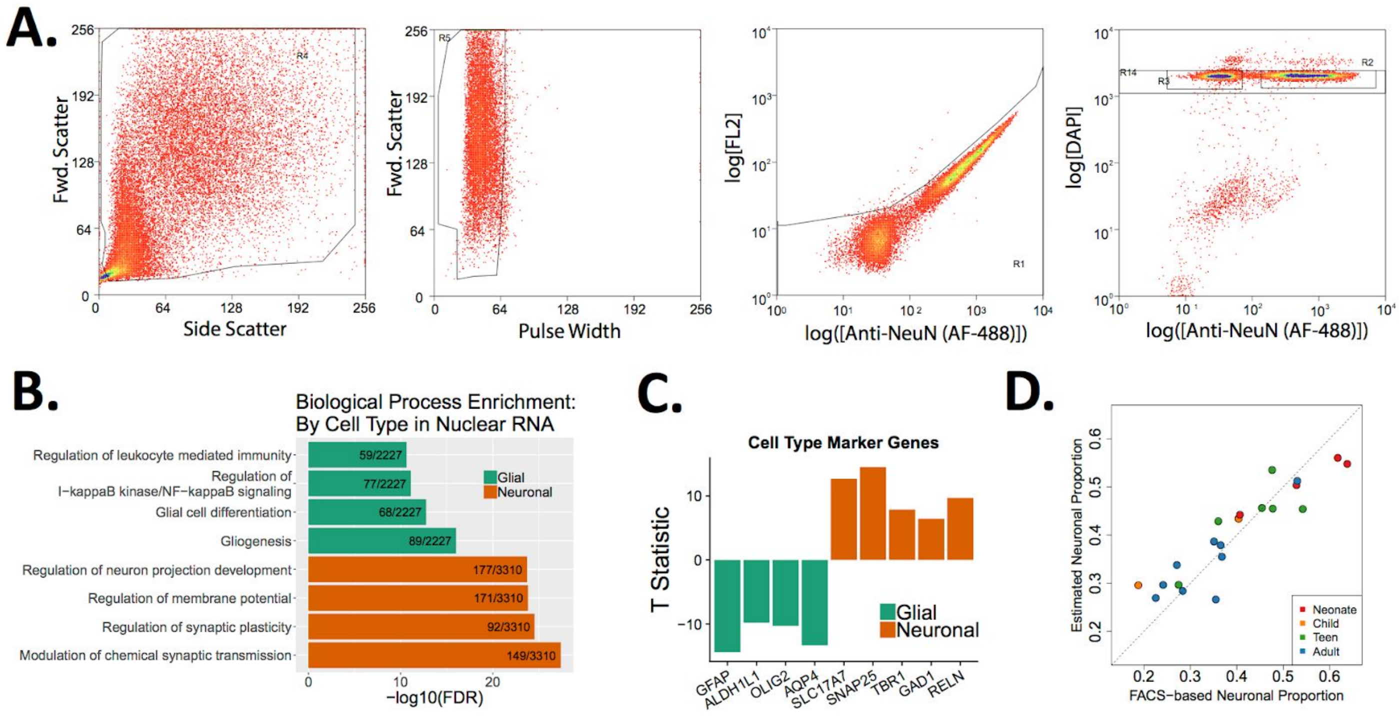

<u>Figure S1: Confirmation of neuronal- and glial-enriched identity of NeuN+ and NeuN- samples.</u> **(A)** Representative gating strategy from fluorescence-activated nuclear sorting by NeuN antibody. Debris is first reduced by selecting events based on forward scatter and side scatter, then aggregates are reduced by measuring pulse width. Autofluorescent events are discarded by measuring true Alexa fluor-488 signal compared to signal in the FL2 channel. Finally, singlets are determined by DAPI staining, and Alexa fluor-488 positive and negative events are collected. **(B)** Top biological processes enriched in genes significantly differentially expressed in nuclear RNA from NeuN+ (labeled "Neurons") and NeuN- (labeled "Glia") at FDR<0.05. **(C)** T statistics for marker genes for neuronal and non-neuronal identity show that the genes are differentially expressed in NeuN+ and NeuN- nuclear RNA (FDR<0.05). **(D)** The estimated proportion of neurons in homogenate WGBS samples based on statistical deconvolution using differentially methylated cytosines between NeuN+ and NeuN- samples is highly correlated with the empirically-derived proportion of NeuN+ events.

Homogenate data: $p_{ij} = \alpha_i + \beta_i^H\ Age_j + \varepsilon_{ij}$

Sorted data: $p_{ij} = \alpha_i + \beta_i^C\ Age_j + \gamma_i\ Type_j + \delta_i\ Age_j * Type_j + \varepsilon_{ij}$

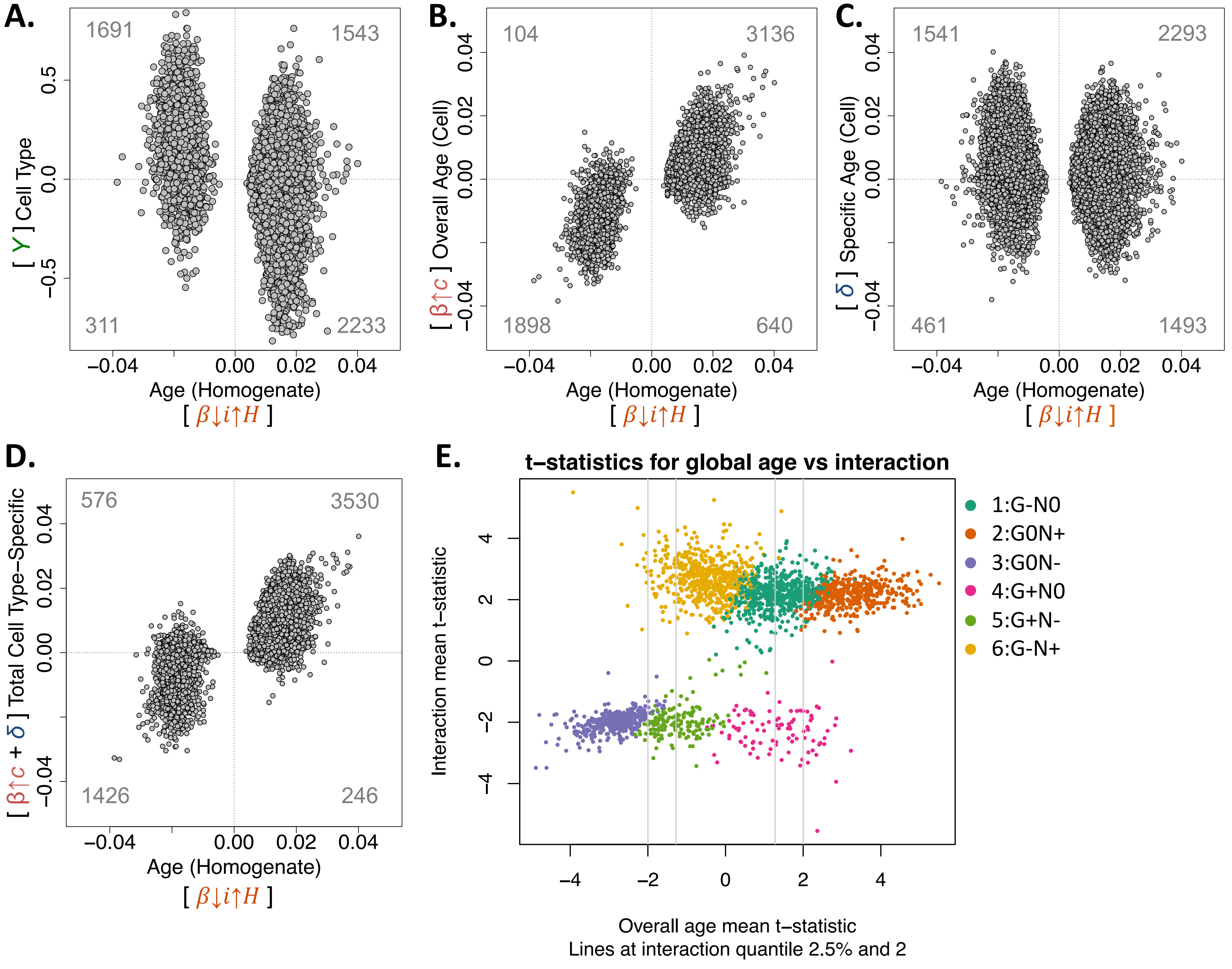

Figure S2: Detecting developmental changes in homogenate vs. cell type-specific DNAm data. **(A)** Developmental age effect coefficients of individual CpGs as measured in homogenate samples compared against cell type effect coefficients in cell type-specific samples after adjusting for age. **(B)** Developmental age effect coefficients of individual CpGs as measured in homogenate samples compared against the developmental effect adjusting for cell type in cell type-specific samples. **(C)** Developmental age effect coefficients as measured in homogenate samples compared against the cumulative age and cell type interaction effect coefficients in cell type-specific samples at the CpG level. **(D)** Developmental age effect coefficients as measured in homogenate samples compared against the estimated cell type-specific age effects for cell type-specific samples at the CpG level. The plot axes are color coded for the coefficients they represent from the model assessing age-associated DNAm changes in homogenate tissue and the model assessing cell type-resolved age changes in the NeuN-sorted data (see above). The number of cytosines in each quadrant are listed in gray. **(E)** Overall age mean t-statistic for the cdDMRs (x-axis) against the mean interaction t-statistic (y-axis) with the 2.5% quantile from the y-axis shown on the x-axis. Colors are the same as those from **Figure 1B**: teal=Group 1 (decreasing glial methylation, increasing neuronal methylation), orange=Group 2 (static glial methylation, increasing neuronal methylation), purple=Group 3 (static glial methylation, decreasing neuronal methylation), pink=Group 4 (increasing glial methylation, static neuronal methylation), green=Group 5 (increasing glial methylation, decreasing neuronal methylation), gold=Group 6 (decreasing glial methylation, static neuronal methylation).

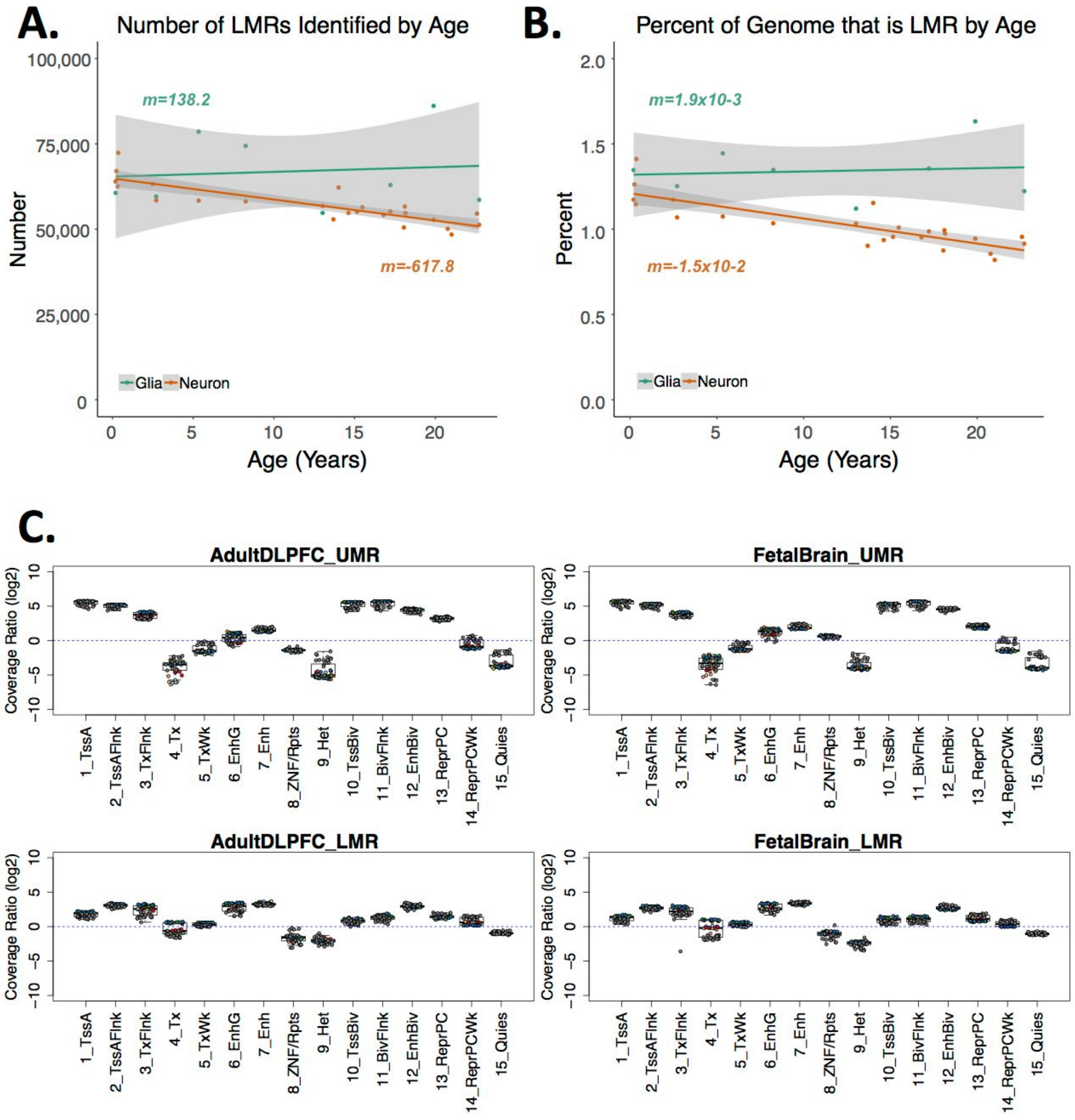

Figure S3: Unmethylated Regions (UMRs) and Low-methylated regions (LMRs). **(A)** Number of LMRs and **(B)** percent of the genome covered by LMRs by age stratified by cell type. Shading indicates the standard error of the linear model. The slope of the linear model for decreasing neuronal LMR number and size in **(A)** and **(B)** are 5.5x and 8.7x that of the glial samples,

respectively. **(C)** Roadmap Epigenomics Consortium predicted chromatin-enriched states for LMRs and UMRs as defined in adult DPLFC and fetal brain. log2(Coverage Ratio) represents the enrichment of the proportion of bases within LMRs or UMRs in a chromatin state compared to the rest of the genome.

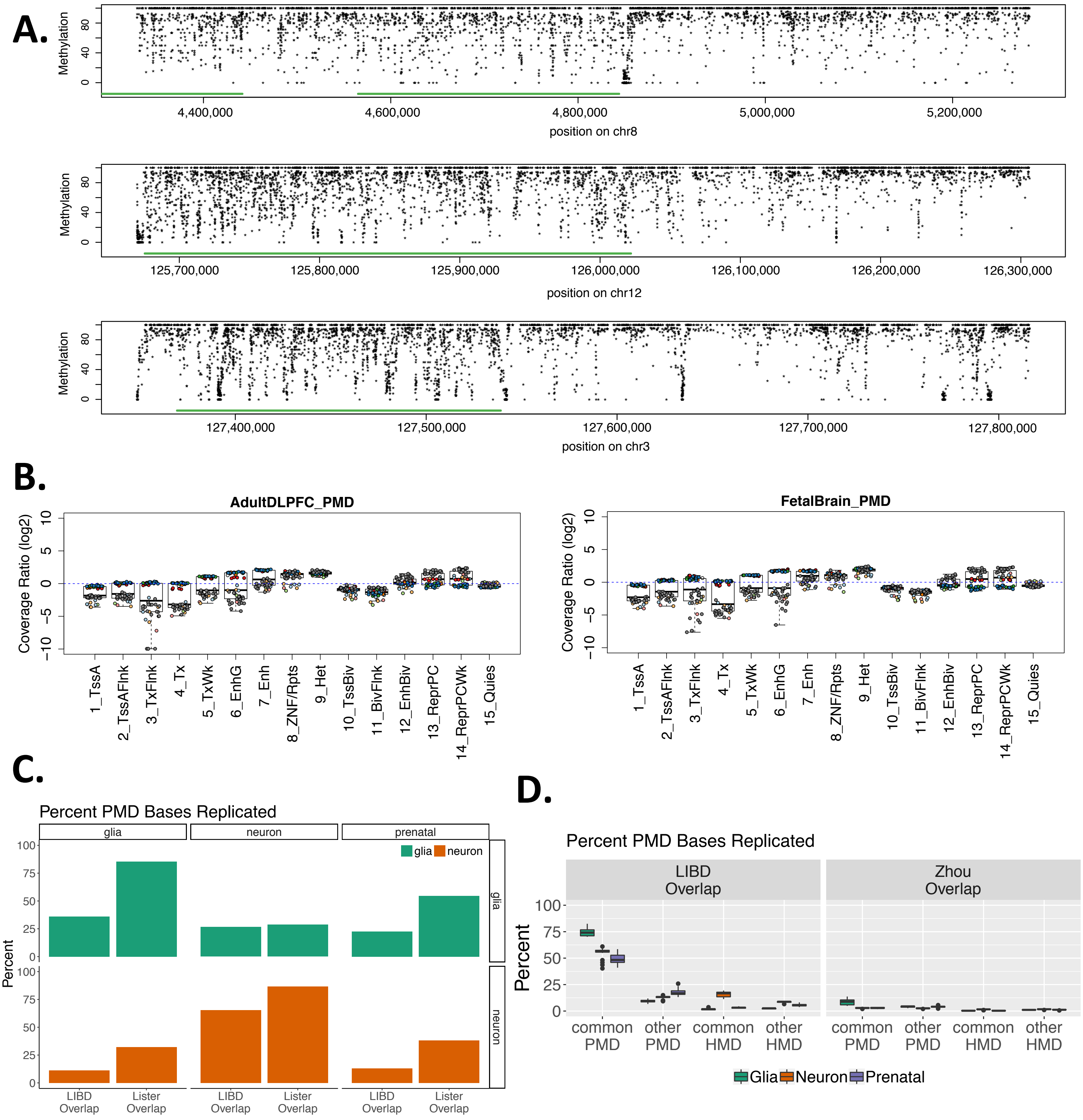
A.
Methylation
position on chr8
4,400,000
4,600,000
4,800,000
5,000,000
5,200,000
position on chr12
125,700,000
125,800,000
125,900,000
126,000,000
126,100,000
126,200,000
126,300,000
position on chr3
127,400,000
127,500,000
127,600,000
127,700,000
127,800,000
B.
AdultDLPFC_PMD
FetalBrain_PMD
Coverage Ratio (log2)
1_TssA
2_TssAFlnk
3_TxFlnk
4_Tx
5_TxWk
6_EnhG
7_Enh
8_ZNF/Rpts
9_Het
10_TssBiv
11_BivFlnk
12_EnhBiv
13_ReprPC
14_ReprPCWk
15_Quies
C.
Percent PMD Bases Replicated
glia
neuron
prenatal
Percent
LIBD Overlap
Lister Overlap
D.
Percent PMD Bases Replicated
LIBD Overlap
Zhou Overlap
Percent
common PMD
other PMD
common HMD
other HMD
Glia
Neuron
Prenatal

Figure S4: Partially methylated domains (PMDs). **(A)** Example PMDs on chromosomes 8, 12, and 3. PMDs are highlighted in green. **(B)** Roadmap Epigenomics Consortium chromatin state enrichment for PMDs. log2(Coverage Ratio) represents the enrichment of the proportion of bases within PMDs in a state compared to the rest of the genome. **(C)** Percent of PMD base-pairs that are replicated in the Lister *et al.* (2013) FANS samples[1]. Rows represent Lister *et al.* (2013) glia and neurons, and columns represent the glia, neurons and prenatal samples from this paper (LIBD). Each bar represents the percent of PMD bases shared between that quadrant's cell types, using either total LIBD or total Lister PMD bases as the denominator. **(D)** Percent of PMD base pairs per sample that are replicated in either common or unique PMDs and high methylated domains (HMDs) identified in Zhou *et al.[2]* Samples are colored based on whether the sample was postnatal neuron, postnatal glia, or bulk prenatal cortex.

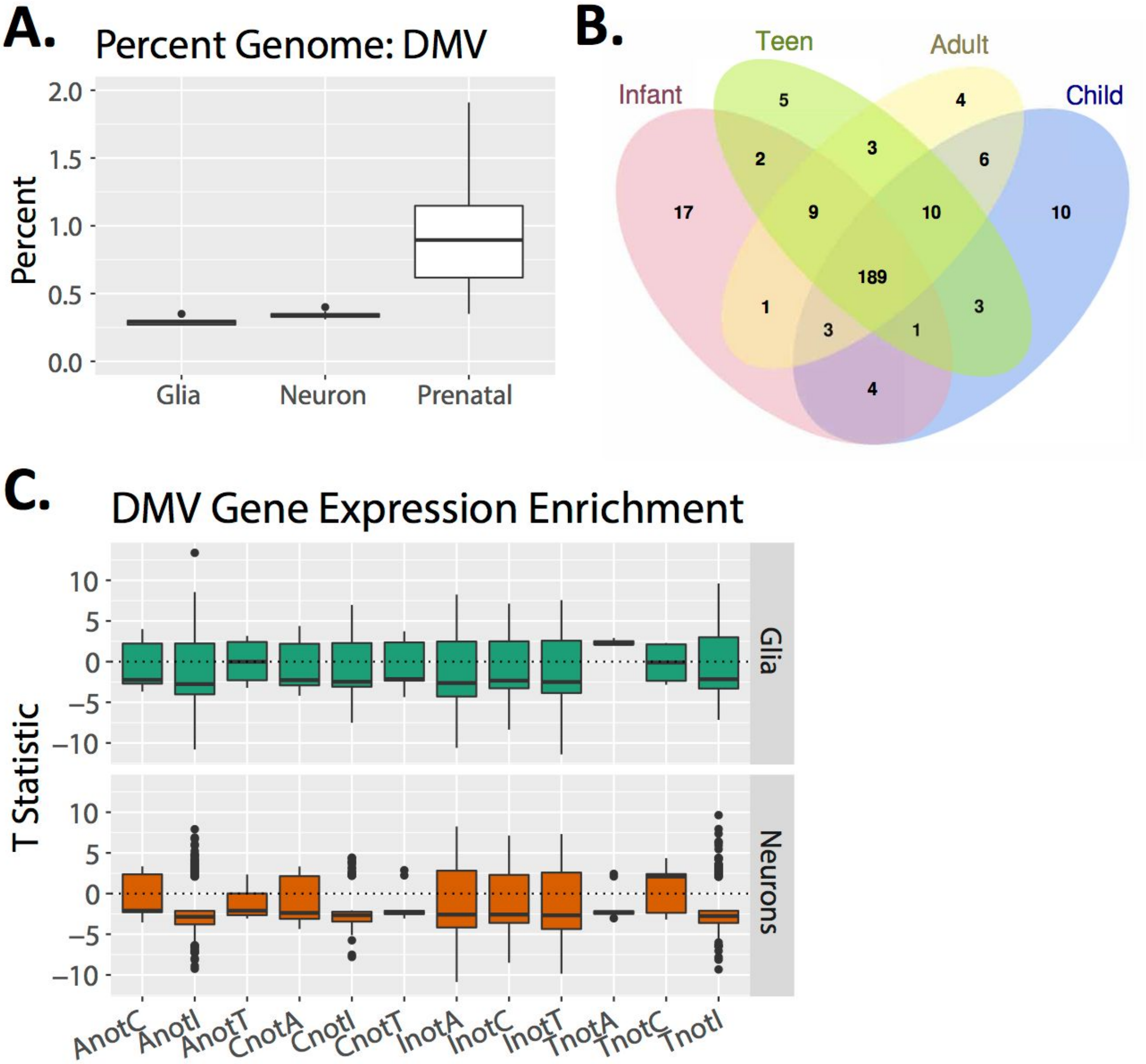

Figure S5: DNA methylation valleys (DMVs). **(A)** Percent of the genome covered by DMVs in postnatal glia and neurons and bulk prenatal cortex. **(B)** Overlap of transcription factor (TF) genes within DMVs by age in neurons. **(C)** Expression enrichment between age groups in TF genes excluded from DMVs in one group but not the other in neurons and glia. A negative T-statistic signifies greater expression in the age group in which the gene is not in a DMV. I=Infant (0-1 years); C=Child (1-10 years), T=Teen (11-17 years) and A=Adult (18+ years).

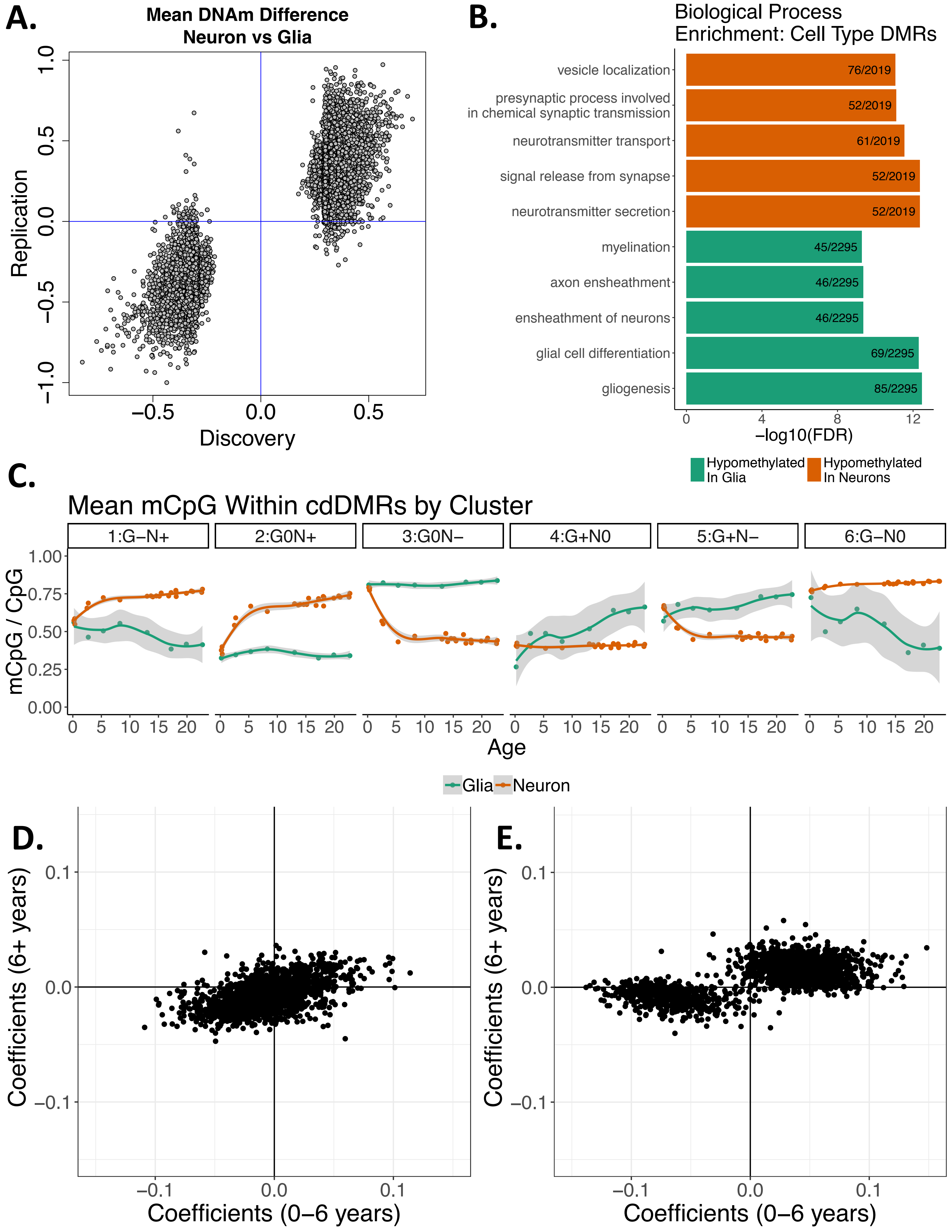
A.
Mean DNAm Difference
Neuron vs Glia
Replication
Discovery
B.
Biological Process
Enrichment: Cell Type DMRs
vesicle localization 76/2019
presynaptic process involved in chemical synaptic transmission 52/2019
neurotransmitter transport 61/2019
signal release from synapse 52/2019
neurotransmitter secretion 52/2019
myelination 45/2295
axon ensheathment 46/2295
ensheathment of neurons 46/2295
glial cell differentiation 69/2295
gliogenesis 85/2295
−log10(FDR)
Hypomethylated In Glia
Hypomethylated In Neurons
C.
Mean mCpG Within cdDMRs by Cluster
1:G−N+
2:G0N+
3:G0N−
4:G+N0
5:G+N−
6:G−N0
mCpG / CpG
Age
Glia
Neuron
D.
Coefficients (6+ years)
Coefficients (0−6 years)
E.
Coefficients (6+ years)
Coefficients (0−6 years)

<u>Figure S6: Differentially methylated regions (DMRs).</u> **(A)** Replication of cell type DNAm differences at the CpG level between neurons and glia (NeuN+ and NeuN- samples) in our and Lister *et al.* data[1]. X and Y axes are linear model coefficients. (**B)** Enriched biological process ontology terms in the DMRs by cell type. The number of genes within each ontology group that overlaps a cell type DMR is listed. (**C)** Mean mCpG within the cdDMRs by cluster from **Figure 1B** across development. Loess line and standard error shading are depicted. **(D)** Coefficients of linear regression on the mean mCpG level per cdDMR in glial samples younger than 6 years and older. **(E)** Coefficients of linear regression on the mean mCpG level per cdDMR in neuronal samples younger than 6 years and older.

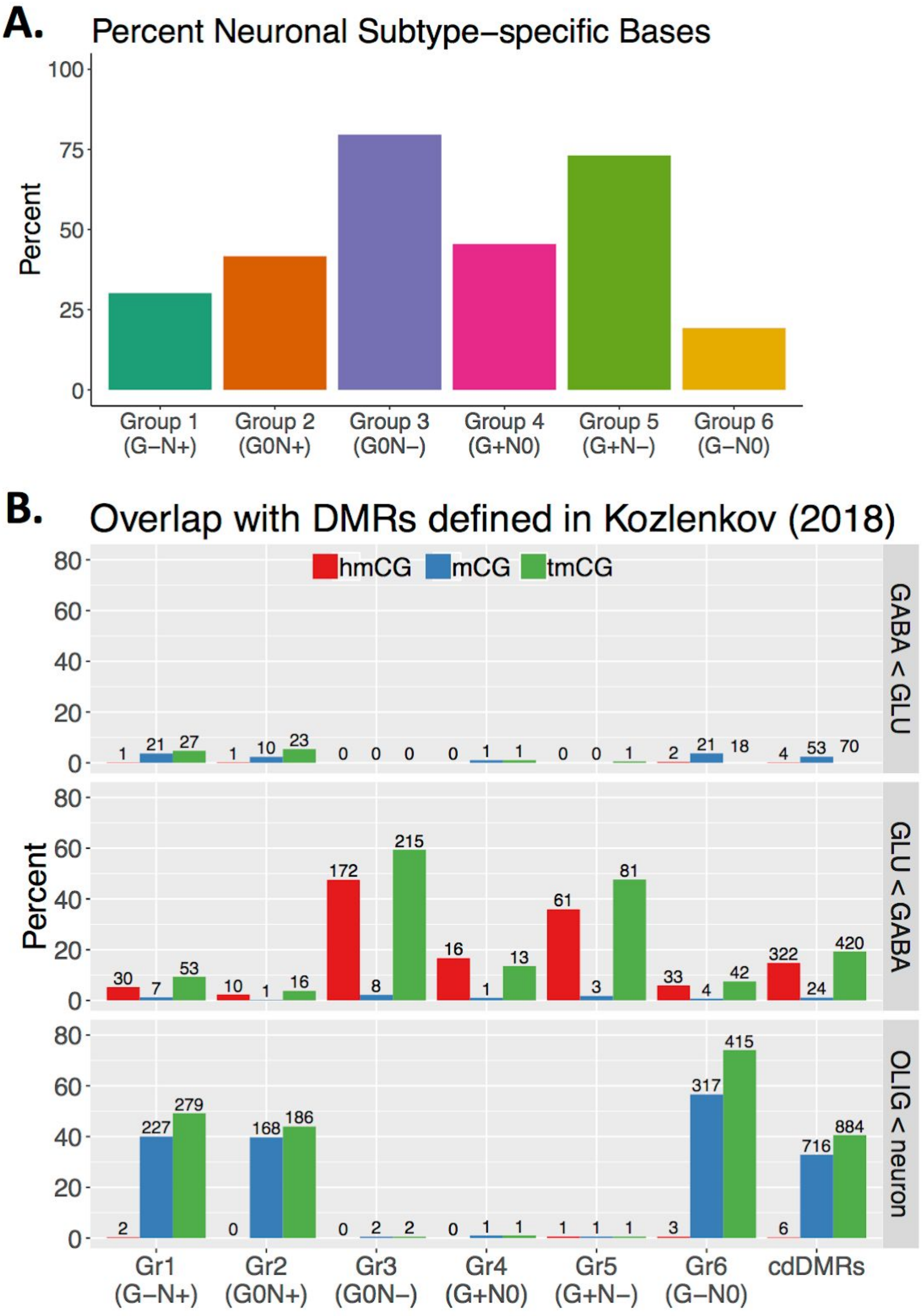
A.
Percent Neuronal Subtype−specific Bases
Percent
Group 1 (G−N+)
Group 2 (G0N+)
Group 3 (G0N−)
Group 4 (G+N0)
Group 5 (G+N−)
Group 6 (G−N0)
B.
Overlap with DMRs defined in Kozlenkov (2018)
hmCG
mCG
tmCG
GABA < GLU
GLU < GABA
OLIG < neuron
Percent
Gr1 (G−N+)
Gr2 (G0N+)
Gr3 (G0N−)
Gr4 (G+N0)
Gr5 (G+N−)
Gr6 (G−N0)
cdDMRs

Figure S7: Cell type-specific developmental DMRs and neuronal subtype methylation and hydroxymethylation. **(A)** Percent of bases within each group of cdDMRs as defined in **Figure 1B** that are differentially methylated by neuronal subtypes in Luo *et al.[3]*. **(B)** Percent of cdDMRs within each of the 6 groups as defined in **Figure 1B** that overlap DMRs of methylcytosine (mC captured using oxidative bisulfite sequencing), hydroxymethylcytosine (hmC), or total cytosine methylation (tmC or mC+hmC, captured using standard bisulfite sequencing) as defined by Kozlenkov *et al.[4]*. The plot is stratified by whether the Kozlenkov *et al.* (2018) DMRs are more highly methylated in GABAergic or glutamatergic neurons, or in overall neurons compared to oligodendrocytes. The number of overlapping cdDMRs are listed above each bar.

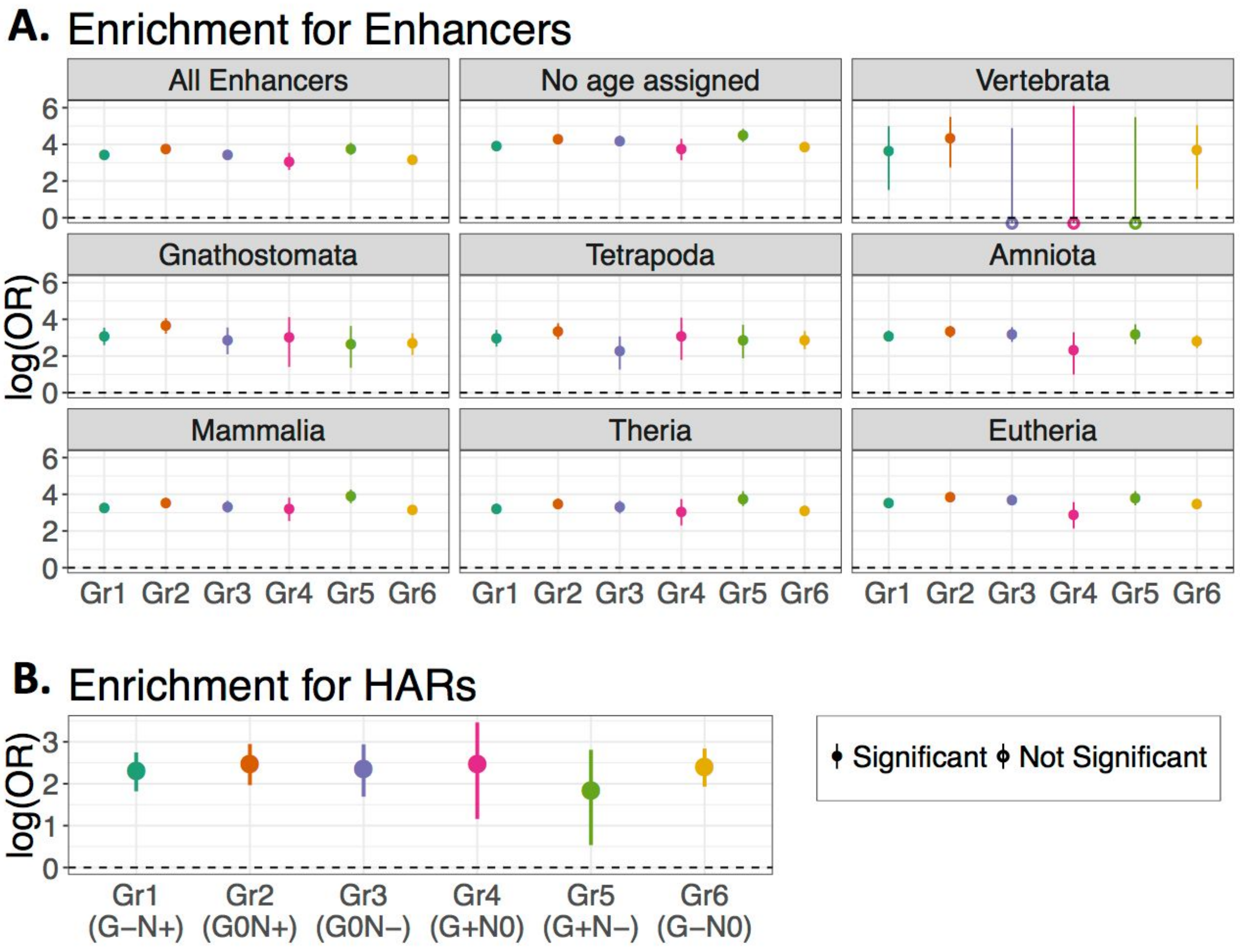

Figure S8: cdDMR overlap of human brain developmental enhancers and Human Accelerated Regions (HARs). **(A)** Enrichment for enhancers in the six clusters of cdDMRs from **Figure 1B** at the CpG-level. **(B)** Enrichment for human accelerated regions (HARs) in the six clusters of cdDMRs from **Figure 1B** at the CpG-level. Filled in circles indicate FDR<0.05, and error bars are the 95% confidence interval for the log(odds ratio).

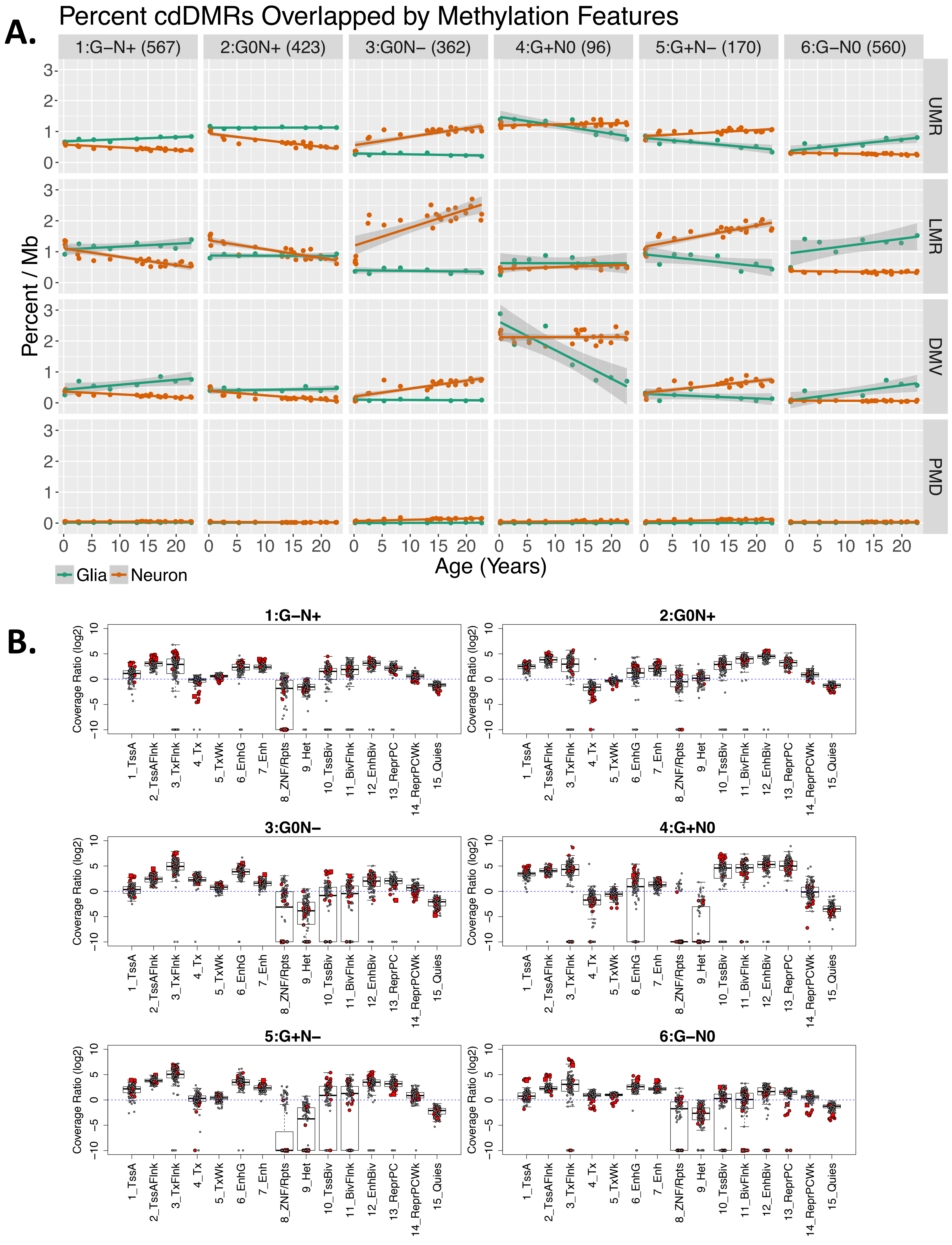
A.
Percent cdDMRs Overlapped by Methylation Features
1:G−N+ (567)
2:G0N+ (423)
3:G0N− (362)
4:G+N0 (96)
5:G+N− (170)
6:G−N0 (560)
UMR
LMR
DMV
PMD
Percent / Mb
Age (Years)
Glia
Neuron
B.
1:G−N+
2:G0N+
3:G0N−
4:G+N0
5:G+N−
6:G−N0
Coverage Ratio (log2)
1_TssA
2_TssAFlnk
3_TxFlnk
4_Tx
5_TxWk
6_EnhG
7_Enh
8_ZNF/Rpts
9_Het
10_TssBiv
11_BivFlnk
12_EnhBiv
13_ReprPC
14_ReprPCWk
15_Quies

Figure S9: Cell type-specific, developmentally dynamic DMRs (cdDMRs) and epigenetic states. **(A)** Percent of cdDMRs by the k-mean groups from **Figure 1B** overlapped by UMR, LMR, DMV and PMD sequence across age. Lines show linear regression and shading indicates the standard error. **(B)** Roadmap Epigenomics Consortium enriched chromatin states for the six clusters of cdDMRs from **Figure 1B** at the CpG-level. log2(Coverage Ratio) represents the enrichment of the proportion of bases within each cdDMR group in a chromatin state compared to the rest of the genome.

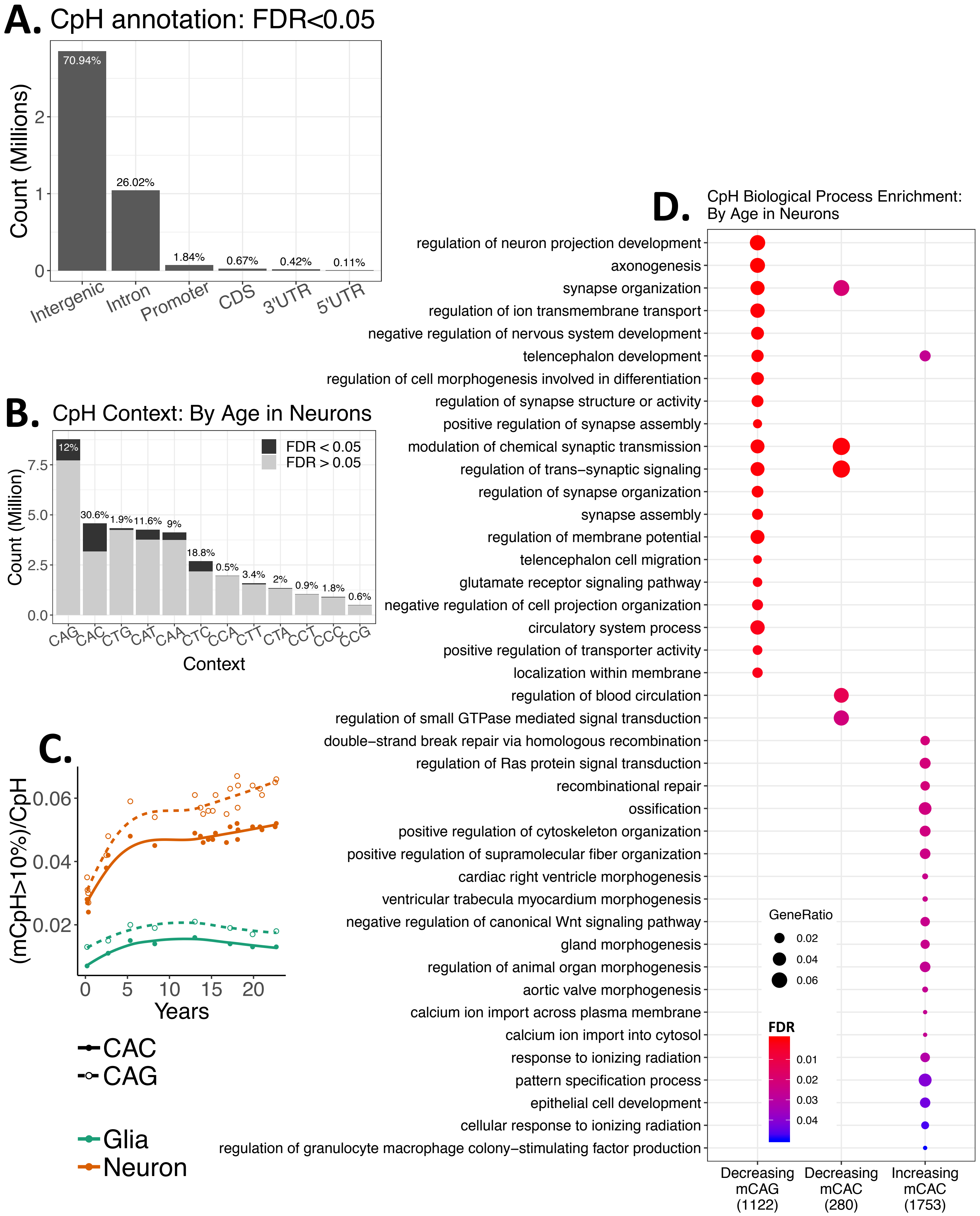
A.
CpH annotation: FDR<0.05
Count (Millions)
70.94%
26.02%
1.84%
0.67%
0.42%
0.11%
Intergenic
Intron
Promoter
CDS
3'UTR
5'UTR
B.
CpH Context: By Age in Neurons
FDR < 0.05
FDR > 0.05
Count (Million)
12%
30.6%
1.9%
11.6%
9%
18.8%
0.5%
3.4%
2%
0.9%
1.8%
0.6%
CAG CAC CTG CAT CAA CTC CCA CTT CTA CCT CCC CCG
Context
C.
(mCpH>10%)/CpH
Years
CAC
CAG
Glia
Neuron
D.
CpH Biological Process Enrichment: By Age in Neurons
regulation of neuron projection development
axonogenesis
synapse organization
regulation of ion transmembrane transport
negative regulation of nervous system development
telencephalon development
regulation of cell morphogenesis involved in differentiation
regulation of synapse structure or activity
positive regulation of synapse assembly
modulation of chemical synaptic transmission
regulation of trans−synaptic signaling
regulation of synapse organization
synapse assembly
regulation of membrane potential
telencephalon cell migration
glutamate receptor signaling pathway
negative regulation of cell projection organization
circulatory system process
positive regulation of transporter activity
localization within membrane
regulation of blood circulation
regulation of small GTPase mediated signal transduction
double−strand break repair via homologous recombination
regulation of Ras protein signal transduction
recombinational repair
ossification
positive regulation of cytoskeleton organization
positive regulation of supramolecular fiber organization
cardiac right ventricle morphogenesis
ventricular trabecula myocardium morphogenesis
negative regulation of canonical Wnt signaling pathway
gland morphogenesis
regulation of animal organ morphogenesis
aortic valve morphogenesis
calcium ion import across plasma membrane
calcium ion import into cytosol
response to ionizing radiation
pattern specification process
epithelial cell development
cellular response to ionizing radiation
regulation of granulocyte macrophage colony−stimulating factor production
GeneRatio
0.02
0.04
0.06
FDR
0.01
0.02
0.03
0.04
Decreasing mCAG (1122)
Decreasing mCAC (280)
Increasing mCAC (1753)

Figure S10: CpH methylation distribution, levels and context-specific biological process ontology.

(A) Number of differentially methylated CpHs by cell type (FDR<5%) falling in different genomic annotations across the genome. Annotation was prioritized CDS > 5'UTR > 3'UTR > Intron > Promoter > Intergenic. **(B)** Breakdown of measured CpH by trinucleotide context. Differentially methylated mCpH by age in neurons (FDR<0.05) are colored in dark gray. **(C)** Accumulation of mCpH by trinucleotide context over development, stratified by cell type, where the y-axis reflects the number of CAC or CAG sites with greater than 10% methylation divided by the total number of CpH sites in each sample. This differs from Figure 2A, where the y-axis reflects the number of methylated CAC or CAG sites divided by the number of CpH sites in that trinucleotide co**ntext (ie, either** (mCAC>10%)/(total CAC sites) or (mCAG>10%)/(total CAG sites). **(D)** The top 20 biological process ontology terms enriched for genes exclusively overlapping CAG and CAC sites with significantly increasing or decreasing methylation levels in neurons over postnatal development (FDR<0.05). That is, decreasing mCAG-overlapping genes shown in **(D)** are those that do not include decreasing mCAC; decreasing mCAC does not include decreasing mCAG; and increasing mCAC does not include increasing mCAG.

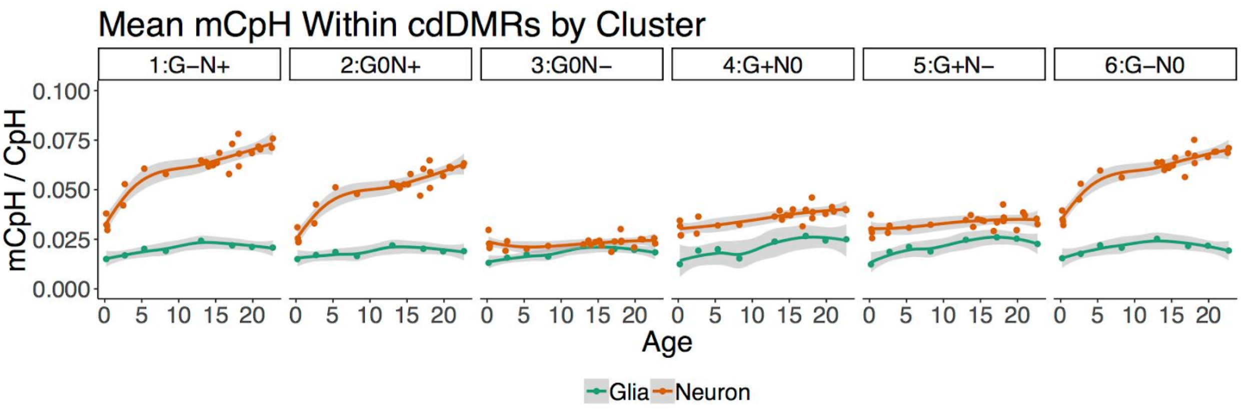

Figure S11: Trajectories of CpH methylation accumulation in cdDMR groups. Mean mCpH within the cdDMRs by cluster from **Figure 1B** across development. Loess line and standard error shading are depicted.

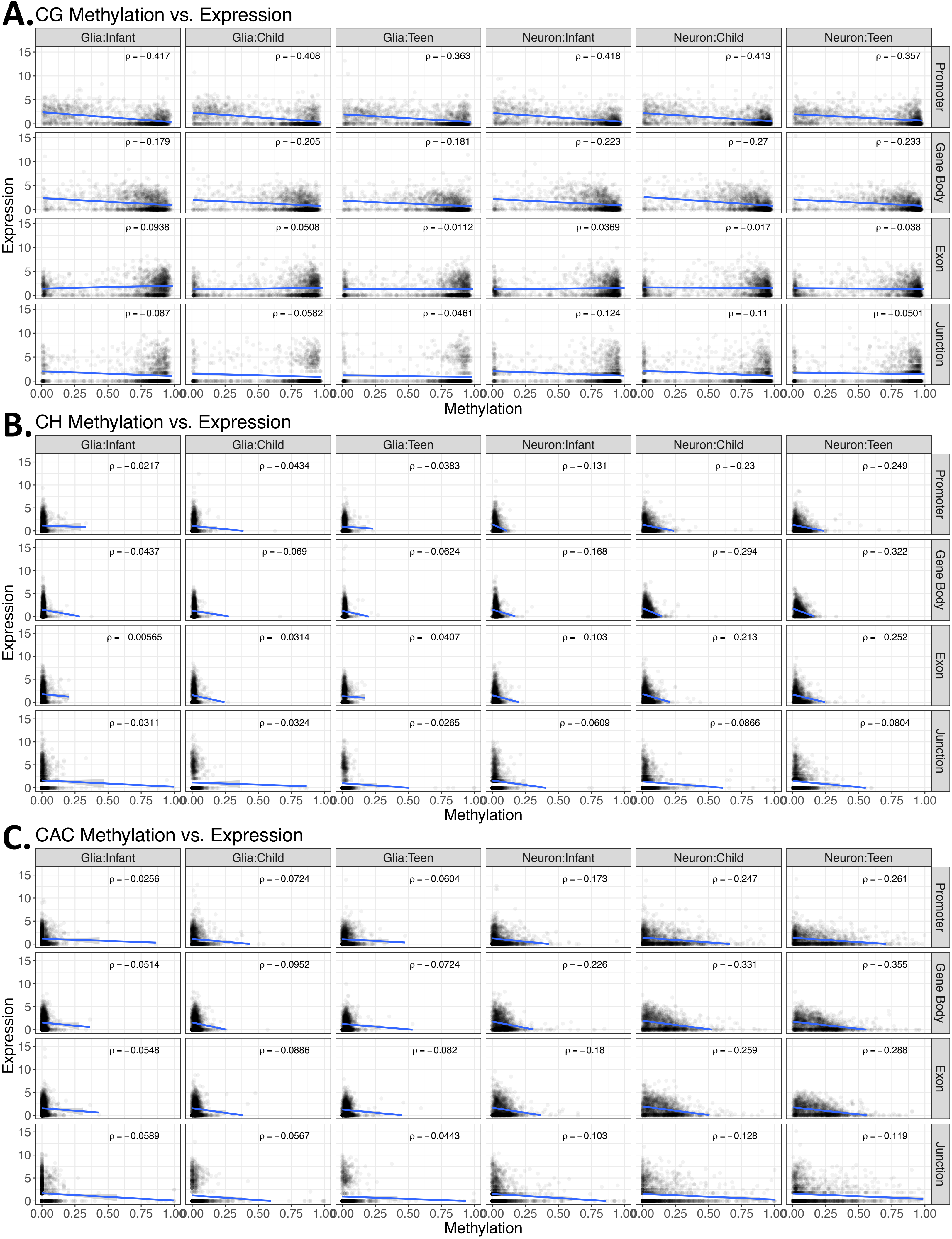
A.
CG Methylation vs. Expression
Glia:Infant
Glia:Child
Glia:Teen
Neuron:Infant
Neuron:Child
Neuron:Teen
Promoter
ρ = −0.417
ρ = −0.408
ρ = −0.363
ρ = −0.418
ρ = −0.413
ρ = −0.357
Gene Body
ρ = −0.179
ρ = −0.205
ρ = −0.181
ρ = −0.223
ρ = −0.27
ρ = −0.233
Exon
ρ = 0.0938
ρ = 0.0508
ρ = −0.0112
ρ = 0.0369
ρ = −0.017
ρ = −0.038
Junction
ρ = −0.087
ρ = −0.0582
ρ = −0.0461
ρ = −0.124
ρ = −0.11
ρ = −0.0501
Expression
Methylation
B.
CH Methylation vs. Expression
Glia:Infant
Glia:Child
Glia:Teen
Neuron:Infant
Neuron:Child
Neuron:Teen
Promoter
ρ = −0.0217
ρ = −0.0434
ρ = −0.0383
ρ = −0.131
ρ = −0.23
ρ = −0.249
Gene Body
ρ = −0.0437
ρ = −0.069
ρ = −0.0624
ρ = −0.168
ρ = −0.294
ρ = −0.322
Exon
ρ = −0.00565
ρ = −0.0314
ρ = −0.0407
ρ = −0.103
ρ = −0.213
ρ = −0.252
Junction
ρ = −0.0311
ρ = −0.0324
ρ = −0.0265
ρ = −0.0609
ρ = −0.0866
ρ = −0.0804
Expression
Methylation
C.
CAC Methylation vs. Expression
Glia:Infant
Glia:Child
Glia:Teen
Neuron:Infant
Neuron:Child
Neuron:Teen
Promoter
ρ = −0.0256
ρ = −0.0724
ρ = −0.0604
ρ = −0.173
ρ = −0.247
ρ = −0.261
Gene Body
ρ = −0.0514
ρ = −0.0952
ρ = −0.0724
ρ = −0.226
ρ = −0.331
ρ = −0.355
Exon
ρ = −0.0548
ρ = −0.0886
ρ = −0.082
ρ = −0.18
ρ = −0.259
ρ = −0.288
Junction
ρ = −0.0589
ρ = −0.0567
ρ = −0.0443
ρ = −0.103
ρ = −0.128
ρ = −0.119
Expression
Methylation

D.

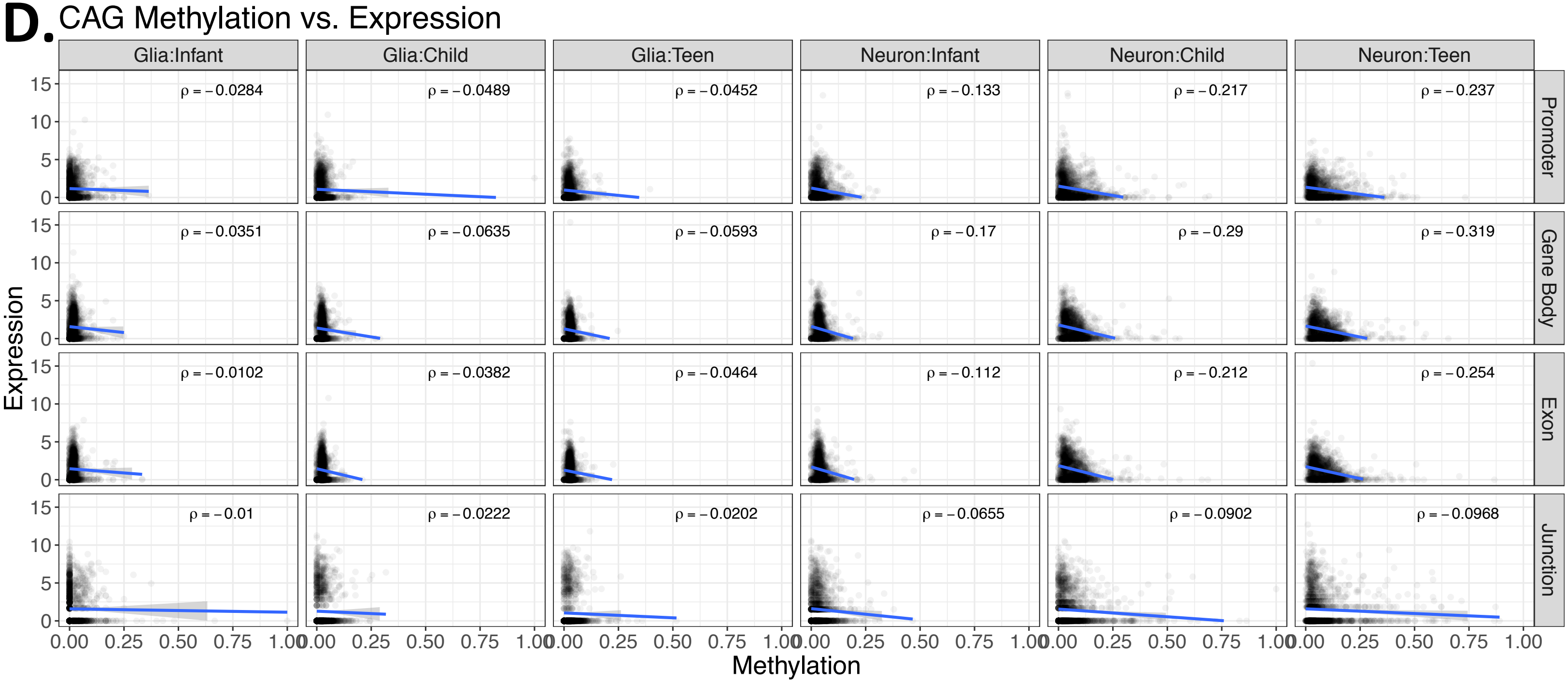
CAG Methylation vs. Expression
Glia:Infant
Glia:Child
Glia:Teen
Neuron:Infant
Neuron:Child
Neuron:Teen
Promoter
Gene Body
Exon
Junction
ρ = −0.0284
ρ = −0.0489
ρ = −0.0452
ρ = −0.133
ρ = −0.217
ρ = −0.237
ρ = −0.0351
ρ = −0.0635
ρ = −0.0593
ρ = −0.17
ρ = −0.29
ρ = −0.319
ρ = −0.0102
ρ = −0.0382
ρ = −0.0464
ρ = −0.112
ρ = −0.212
ρ = −0.254
ρ = −0.01
ρ = −0.0222
ρ = −0.0202
ρ = −0.0655
ρ = −0.0902
ρ = −0.0968
Expression
Methylation

E.

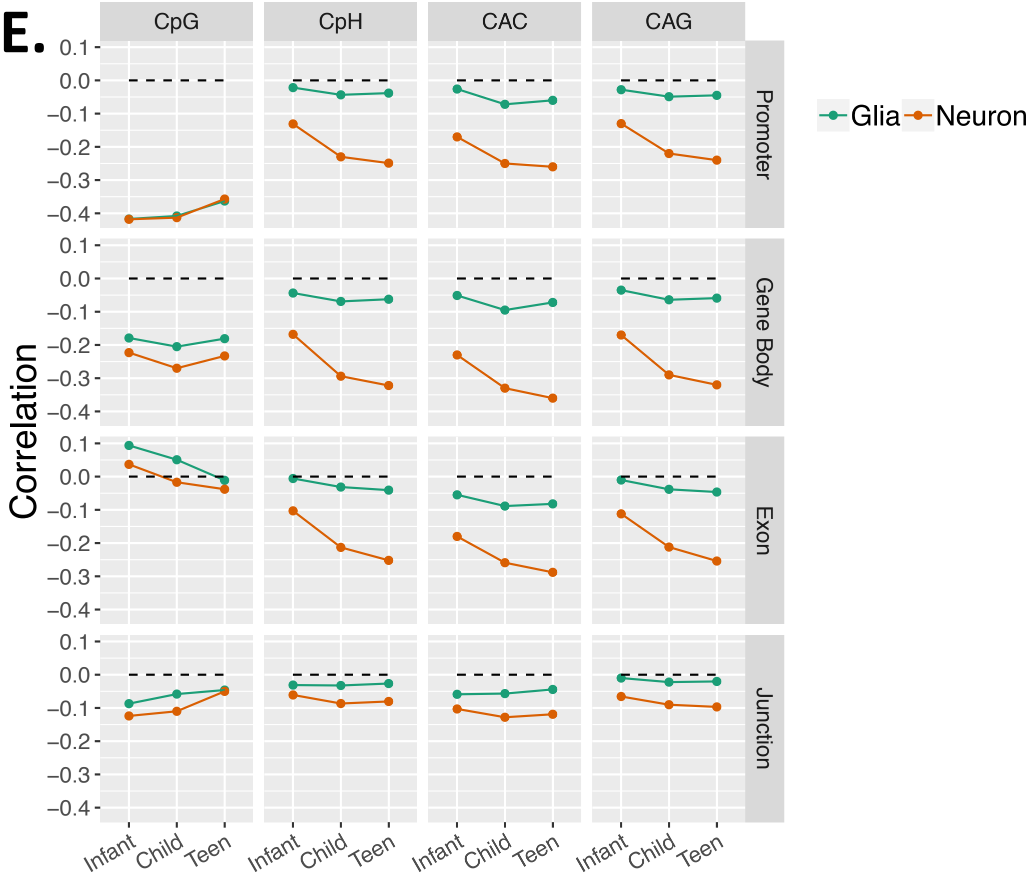
CpG
CpH
CAC
CAG
Promoter
Gene Body
Exon
Junction
Correlation
Infant
Child
Teen
Glia
Neuron

<u>Figure S12: Relationship between methylation and expression.</u> **(A-D)** A random sample of 10,000 expression feature:methylation pairs stratified by age and cell type in columns and feature type in rows. Each dot represents the mean methylation level of **(A)** CpGs, **(B)** CpHs, **(C)** CACs and **(D)** CAGs within the feature across all samples of that age and cell type on the x-axis and the log2([mean FPKM]+1) for the promoters, gene bodies, and exons and the log2([mean junction-overlapping reads per 10 million mapped reads]+1) for the junctions on the y-axis. 10,000 pairs were chosen to reduce overplotting. Linear regression with shaded standard error and the rho for each correlation is listed for each plot. **(E)** Correlation of feature expression and methylation stratified by cytosine context (columns) and feature type (rows).

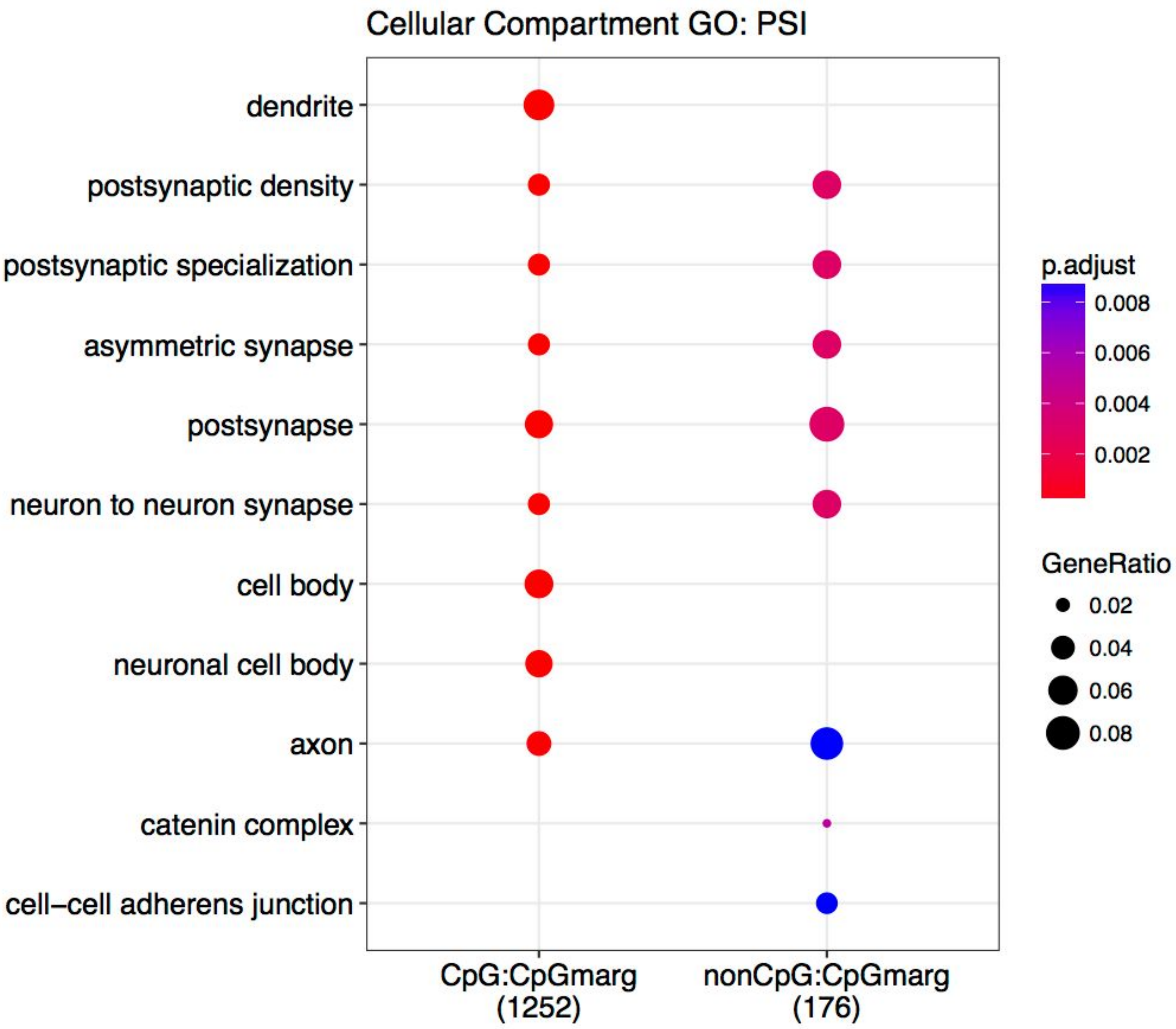

Figure S13: Cellular compartment ontology. Genes containing splicing events as measured by "percent spliced in" (PSI) that are associated with changing CpG and CpH methylation are enriched for cellular compartment gene ontology terms relating to neuronal features.

LIBD WGBS Expression explorer   home   methylation data   methylation summary   documentation

The following table includes all the CpG and nonCpG associations with nearby features at FDR 5%. The features levels are gene, exon, splicing events that affect percent splicing in (PSI).

Any filters applied here affect the results of the methylation summary tab.

Show 10 entries   Search:

i	feature	meth_type	feature_id	symbol	gene_type	meth_coefficient	meth_statistic	meth_FDR	n_samples_with_meth_not0	n_samples_with_m
	Al	All	All	All	All	All	All	All	All	All
1	gene	CpG	ENSG00000258691.1_2	RP11-404P21.8	none	-0.0561	-29.5	6.72e-13	22	
1,2	psi	CpG	ENSG00000129422.14_2	MTUS1	neuron	-0.00112	-26.3	7.97e-13	22	
1,2	psi	CpG	ENSG00000129422.14_2	MTUS1	neuron	0.00112	26.3	7.97e-13	22	
3,4	psi	CpG	ENSG00000196352.14_1	CD55	none	-0.0563	-23.4	7.54e-12	21	
3,4	psi	CpG	ENSG00000196352.14_1	CD55	none	0.0563	23.4	7.54e-12	21	
5	psi	CpG	ENSG00000171724.2_2	VAT1L	none	0.0185	22.5	1.52e-11	22	
6	psi	CpG	ENSG00000145982.11_2	FARS2	none	0.041	21.8	2.73e-11	22	
7	psi	CpG	ENSG00000145982.11_2	FARS2	none	0.0408	21.1	5.02e-11	22	
8	psi	CpG	ENSG00000145526.11_2	CDH18	glia	0.0989	20.7	6.86e-11	22	
9,10	psi	CpG	ENSG00000018625.14_1	ATP1A2	neuron	-0.0216	-20.1	1.21e-10	22	

Showing 1 to 10 of 81,243 entries   Previous 1 2 3 4 5 … 8125 Next

Download methylation data

Association details

Here you can explore in further detail a particular methylation and expression association. Choose the feature type, the methylation type (CpG or nonCpG) and select which association id (from column **i** in previous table) you want to explore.

You can also select a row in the table above and the options will be chosen for you automatically. The i chosen will be the first one in the row.

Feature: gene

Methylation type: nonCpG

Result (association id from column i above): 1

Max i is 29654

Export to UCSC Genome Browser

Methylation vs expression association plot:

Scatter plot comparing methylation vs expression. Samples are colored by age group:

Red: infant, orange: child, green: teen, blue: adult. Details in documentation tab.

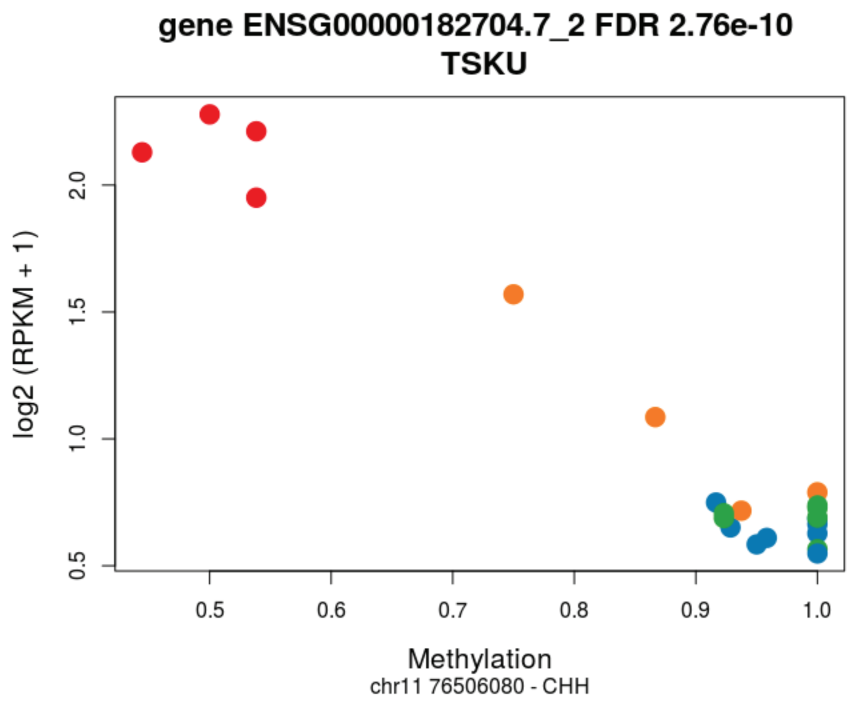

A small amount of jitter has been added to the data to minimize overplotting.

Save image to a PDF file

Expression feature information

```
GRanges object with 1 range and 10 metadata columns:
                      seqnames               ranges strand |    Length           gencodeID       ensemblID
                         <Rle>            <IRanges>  <Rle> | <integer>         <character>     <character>
  ENSG00000182704.7_2    chr11 [76493295, 76509198]      + |      4131 ENSG00000182704.7_2 ENSG00000182704
                         gene_type      Symbol  EntrezID       Class meanExprs     NumTx
                       <character> <character> <integer> <character> <numeric> <integer>
  ENSG00000182704.7_2 protein_coding        TSKU     25987       InGen  3.136563         5
                                                                                                  gencodeTx
                                                                                                <character>
  ENSG00000182704.7_2 ENST00000533752.1_1;ENST00000612930.1_1;ENST00000333090.4_1;ENST00000525167.1_1;ENST00000527881.1_1
  -------
  seqinfo: 36 sequences from an unspecified genome; no seqlengths
```

Download to an Rdata file

Cytosine information

```
GRanges object with 1 range and 2 metadata columns:
      seqnames               ranges strand | c_context trinucleotide_context
         <Rle>            <IRanges>  <Rle> |     <Rle>                 <Rle>
  [1]    chr11 [76506080, 76506080]      - |       CHH                   CAC
  -------
  seqinfo: 25 sequences from an unspecified genome; no seqlengths
```

Download to an Rdata file

For more information check the documentation tab.

Bookmark...

Data license

The data in **LIBD WGBS Expression explorer** is licensed under CC BY 4.0. The legal text can be found here.

Acknowledgements

This research was supported by NIH R21MH102791-01A1 and the Lieber Institute for Brain Development. We thank the Department of Biostatitics at Johns Hopkins Bloomberg School of Public Health for hosting our application on their shinyapps account.

LIEBER INSTITUTE for BRAIN DEVELOPMENT
MALTZ RESEARCH LABORATORIES

JOHNS HOPKINS BLOOMBERG SCHOOL of PUBLIC HEALTH

Figure S14: Web meQTL browser display. Interactive display of the CpHs and CpGs associated with expression at FDR<5% as shown at https://jhubiostatistics.shinyapps.io/wgbsExprs/. This screenshot shows the top nonCpG (mCpH) meQTL association at the gene expression level. Information about the gene is shown under *expression feature*, and information of the methylated C is shown under *cytosine*.

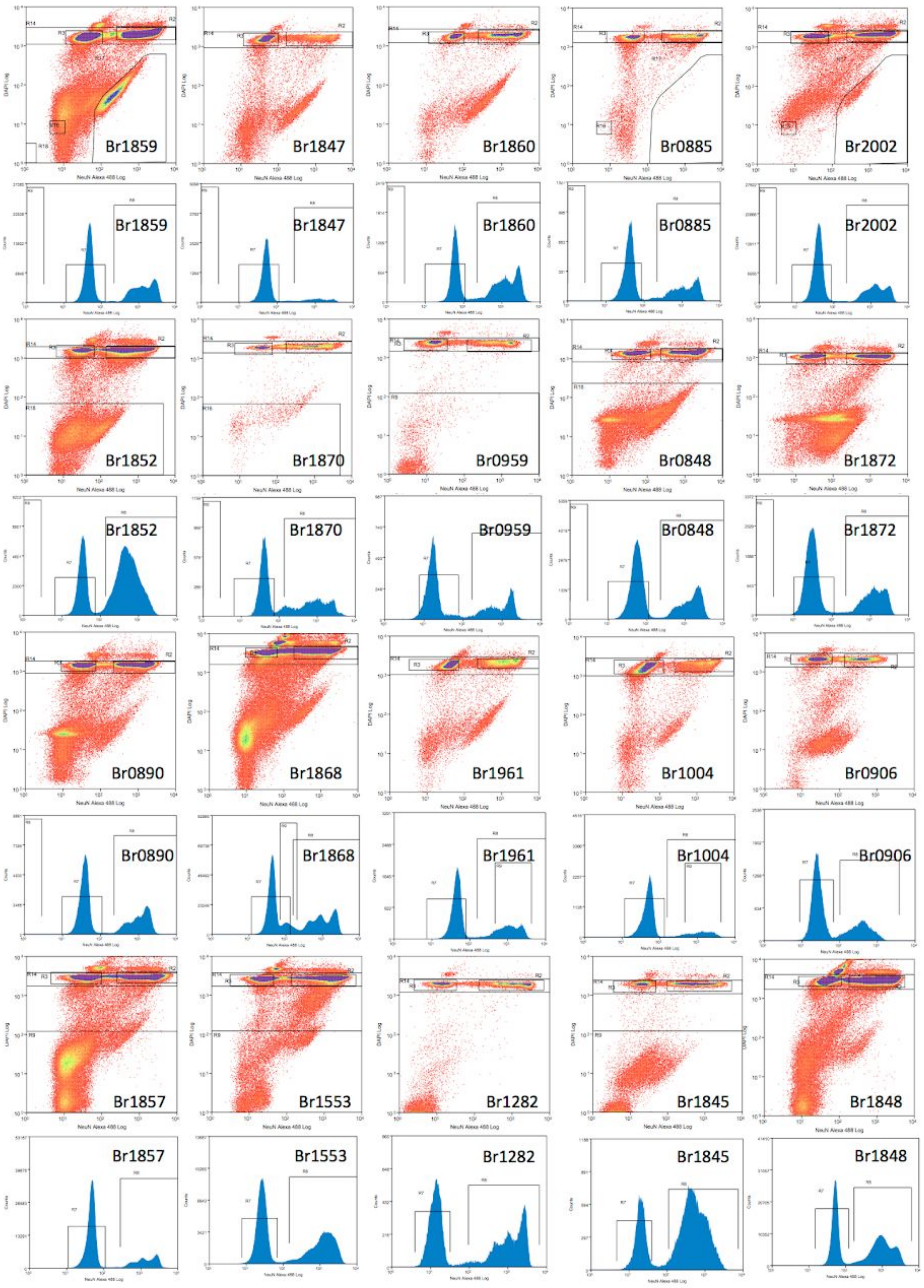
Br1859
Br1847
Br1860
Br0885
Br2002
Br1859
Br1847
Br1860
Br0885
Br2002
Br1852
Br1870
Br0959
Br0848
Br1872
Br1852
Br1870
Br0959
Br0848
Br1872
Br0890
Br1868
Br1961
Br1004
Br0906
Br0890
Br1868
Br1961
Br1004
Br0906
Br1857
Br1553
Br1282
Br1845
Br1848
Br1857
Br1553
Br1282
Br1845
Br1848

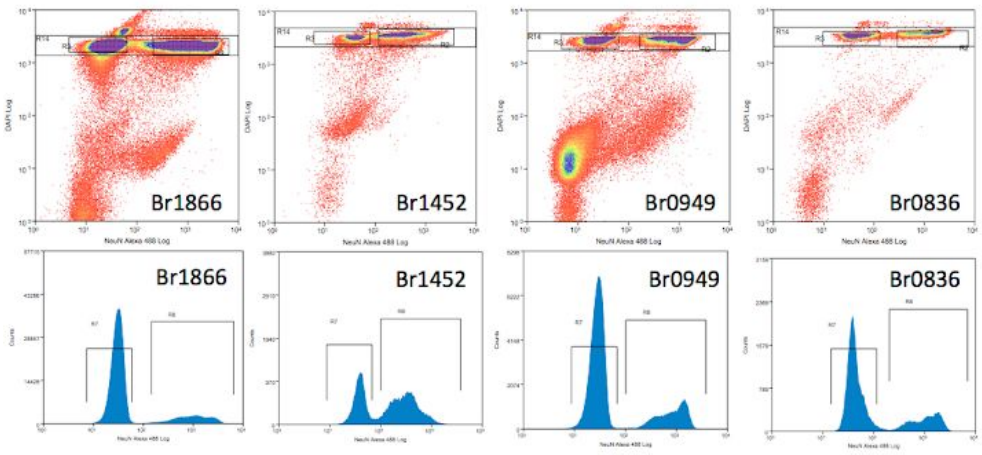

Figure S15: Raw sort data. Raw data collected from the MoFlo Legacy (Beckman Coulter) using Summit (version 4.3) software. The density plots show DAPI signal on the y-axis and Alexa Fluor 488-NeuN signal on the x-axis for events filtered via gates 1-3 as depicted in Figure S1A. The histograms show the distribution of Alexa Fluor 488-NeuN signal after including the gate for singlets based on DAPI signal (gate R14 in the raw data).

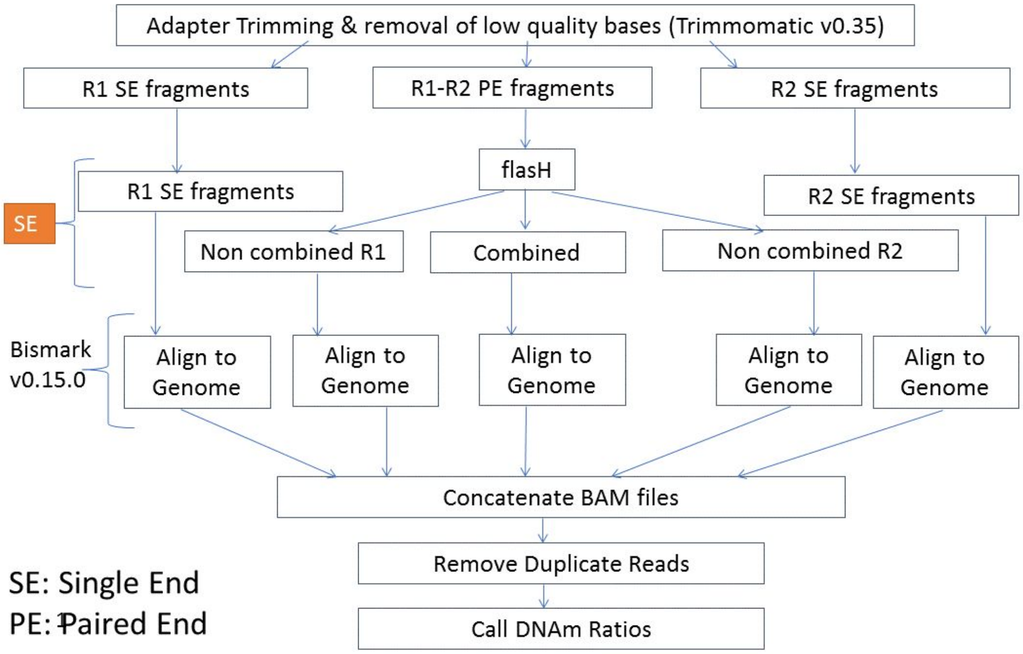

Figure S16: Data processing/alignment pipeline. Overview of the processing steps taken to prepare the WGBS data.

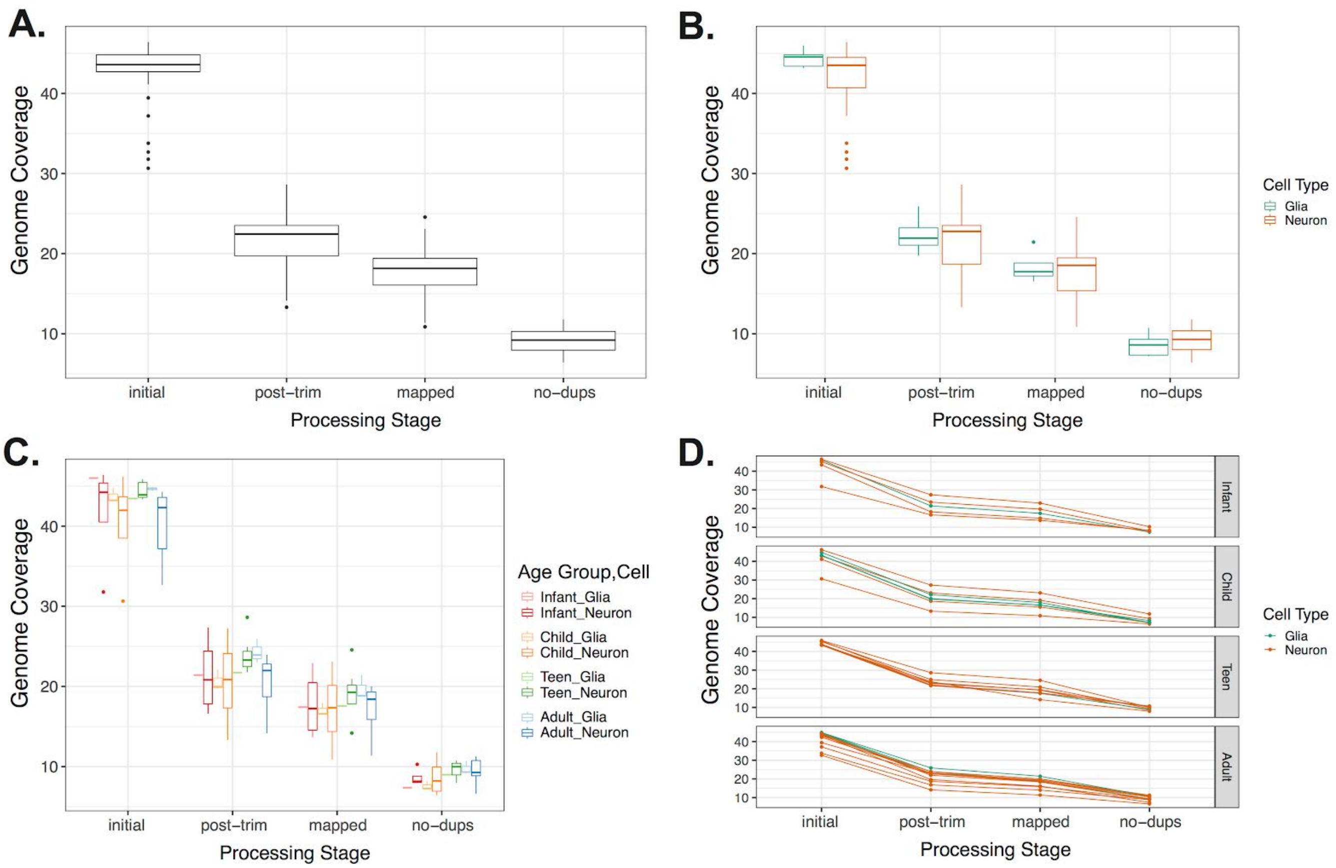

Figure S17: Genome coverage across processing stages. **(A)** Genome coverage across the four main processing stages: initial FASTQ files, after trimming, after mapping to the genome, and after removing duplicated reads. **(B)** Same as (A) but with samples separated by cell type. **(C)** Genome coverage across cell types and four age categories: infant, child, teen and adult. **(D)** Genome coverage trajectories for each sample separated by age category and colored by cell type.

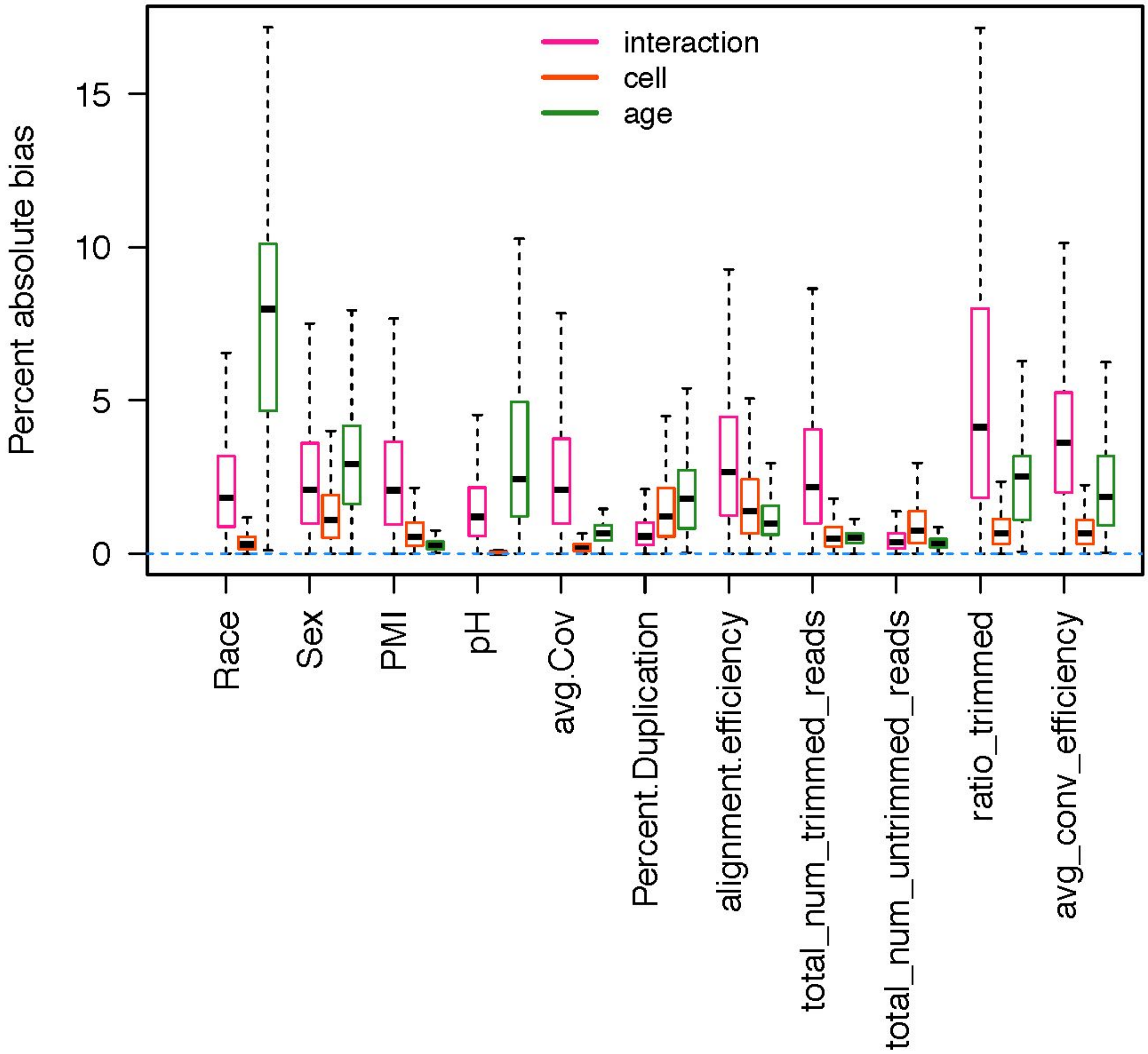

Figure S18: DMR sensitivity analyses. This figure shows the percent absolute bias for the three coefficients of interest used to define the DMRs: age (adjusting for cell type), cell type (adjusting for age), and the interaction between age and cell type. For each DMR type we computed the coefficients after adjusting for each of the covariates in the X-axis (β_{adj}) and computed the percent absolute bias: 100 * | β_{adj} - $\beta_{original}$ | / | $\beta_{original}$ |. All covariates considered show less than 10% absolute bias. The covariates considered are: race, sex, PMI, pH, average coverage, duplication percent, alignment efficiency, total number of trimmed reads, total number of untrimmed reads, ratio of trimmed reads, and average conversion efficiency (lambda).

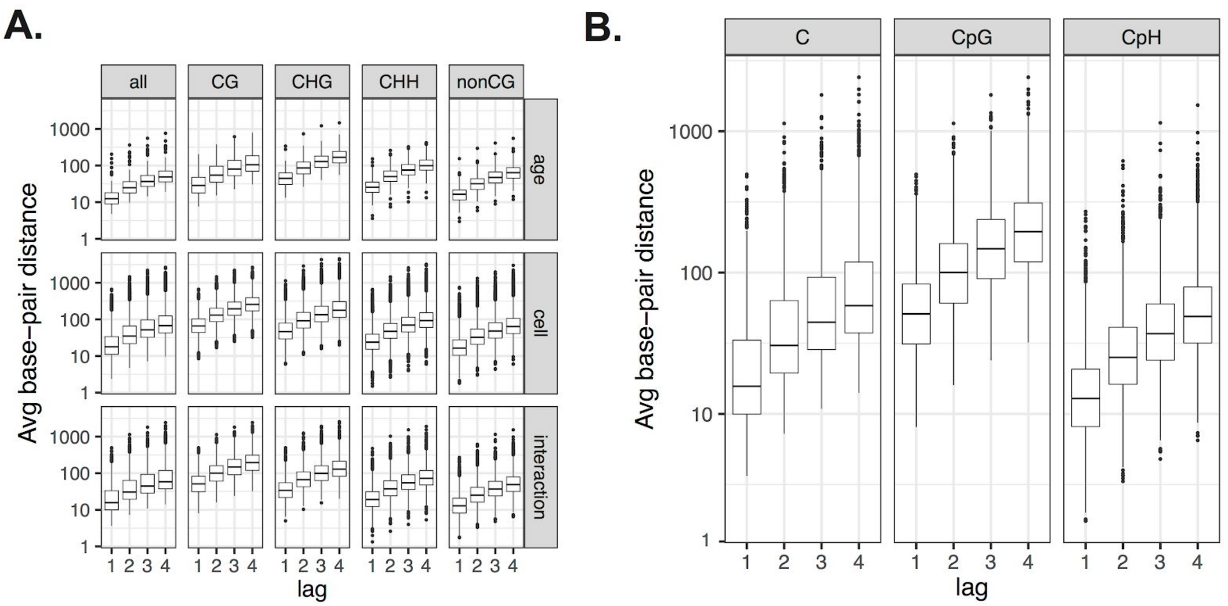

<u>*Figure S19: genome distance versus autocorrelation lag.*</u> For each of the cytosines considered for the autocorrelation analysis in **Figure 2B**, we computed the genome base-pair distance and averaged it for each group of cytosines. **(A)** Average genomic distance for each of the three DMR models and for various contexts of cytosines. **(B)** Average genome distance for the interaction DMRs (cdDMRs) for all cytosines, CpG and CpH. The genome distance is proportional to the lag. This panel is the exact complement to **Figure 2B**.

References

1. Lister R, Mukamel EA, Nery JR, Urich M, Puddifoot CA, Johnson ND, et al. Global epigenomic reconfiguration during mammalian brain development. Science. 2013;341:1237905. doi:10.1126/science.1237905.

2. Zhou W, Dinh HQ, Ramjan Z, Weisenberger DJ, Nicolet CM, Shen H, et al. DNA methylation loss in late-replicating domains is linked to mitotic cell division. Nat Genet. 2018;50:591–602. doi:10.1038/s41588-018-0073-4.

3. Luo C, Keown CL, Kurihara L, Zhou J, He Y, Li J, et al. Single-cell methylomes identify neuronal subtypes and regulatory elements in mammalian cortex. Science. 2017;357:600–4. doi:10.1126/science.aan3351.

4. Kozlenkov A, Li J, Apontes P, Hurd YL, Byne WM, Koonin EV, et al. A unique role for DNA (hydroxy)methylation in epigenetic regulation of human inhibitory neurons. Sci Adv. 2018;4:eaau6190. doi:10.1126/sciadv.aau6190.
