# Supplementary material for: Divergent neuronal DNA methylation patterns across human cortical development reveal critical periods and a unique role of CpH methylation
Source: Genome Biol. 2019 Sep 26;20:196. doi: 10.1186/s13059-019-1805-1 (PMC6761727; doi:10.1186/s13059-019-1805-1)
Supplement: Supplementary file 3 — Review history. (DOCX 22 kb) [file 13059_2019_1805_MOESM3_ESM.docx]

Review History

**First round of review**

**Reviewer 1**

**Are you able to assess all statistics in the manuscript, including the appropriateness of statistical tests used?**

Yes, and I have assessed the statistics in my report.

**Comments to author:**

This paper provides the DNA methylation landscape of human neocortical development, and authors performed functional and phenotype associated studies to indicate the functions of methylation. Especially, they indicated CpH methylation exclusively regulate transcripts and their splicing events. I think this work is interesting. Here are my comments.

1.     All figures are too low resolution to recognize the numbers on the axis, especially the supplementary figures.

2.     In Table S1 and S2, the brain IDs are not matched for some samples, such as Br906 in S2 while Br0906 in S1.

3.     We know that SNPs at CpG / CpH will interfere with methylation calls (PMID: 28822795), and we usually need to remove the know SNPs when we process the WGBS data. However, it is absent in your processing pipeline. I am wondering how many SNPs in your 18.7 million CpGs and 58.1 million CpHs and how they affect your results? In addition, for the coverage threshold of CpG and CpH, why you set different values (3 for CpG and 5 for CpH)?

4.     In Table S3, why the number of CpGs in homogenate samples is almost 1.5 fold of that in Neuron/Glia/Fetal samples while the number of CpHs are same? Additionally, why no methylation level statistics of CpH in homogenate samples?

5.     You did the enrichment analysis of Glia and Neuron specific DE genes on marker genes for neuronal and non-neuronal identity. How to select these marker genes?

6.     For Figure S1A, B, C, and D, in my opinion, the axis titles are not very clear since the number (labeled on the axis) are effect coefficients rather than actual age for the x-axis and cell type for the y-axis. And, how to explain no zero (or approximately zero) age effect coefficients of individual CpGs as measured in homogenate samples?

7.     To clearly show LMRs and UMRs in different samples, could you make a supplementary table to show the number of LMRs and UMRs you detected? You mentioned, "Compared to prenatal homogenate cortex and postnatal glial cells, postnatal neurons showed a general accumulation of mCpG, at a rate 50% faster than the other cells." In section "DNAm as a map of putative functional genomic states". However, from Figure S3A and B, it is hard to get "50% faster", can you show it clearly? From Figure S3C, UMR and LMR showed similar enrichment in both adult and fetal brain, why you just emphasize LMR in manuscripts?

8.     For Figure S5B, most of DMV associated TF genes (189) are overlapped across all four age groups, it seems that DMV overlapped TF genes in an age-independent manner rather than an age-dependent manner. How to explain this? For Figure S5C, the color Glia and Neurons are different from other Figures (like Figure S4C), please make it consistency.

9.     For Figure 1A, we can clearly see the methylation pattern of Infant Neuron is significantly different from others, where infant is defined as 0-1 years. For Figure S6C and D, we can see the range of coefficients in 0-6 years samples are larger than that in 6+ years samples in both glia and neuron. Based on these results, I think, it is hard to get the conclusion of "largest changes occurring in the first five years". In my opinion, you should make a similar analysis like Figure S6C and D for other age segments, like 0-1 years, 0-2 years, to clearly show the methylation changes during development.

10.  For Supplementary Table 5 the CpH context, how to calculate the "%Significant"? Because 7,682,075 / 58,100,000 = 13.22%, 3,194,618 / 58,100,000 = 5.50%, and 4,020,371 / 58,100,000 = 6.92% are different from what you showed in the table, where 58,100,000 is the number of CpH (Supplementary Table 3). You mentioned in manuscript "98.9% of 7,682,075 differentially methylated CpHs were in neurons, at FDR < 5%". I'm wondering if that means 7,597,572 (7 682 075 * 0.989) CpHs show hypomethylated in neurons? Please state it clearly in the manuscript.

11.  I cannot find any information to indicate "first five years of postnatal life—similarly to mCpG—followed by a tapered global increase into adulthood" from Figure S10B. Both Figure S10C and Figure 2A show the proportions of mCpH changes with age increasing, however, the results are totally different and the statements in manuscript and figure legends are different. Please explain why. Figure S10D show 1,124 decreasing mCAG genes, 280 decreasing mCAC genes, and 1,750 increasing mCAC genes, however, in manuscript, you said "3,286 and 1,744 genes that contained significantly increasing and decreasing mCAC versus mCAG over development, respectively", why they are different?

12.  The RNA-seq data was pair-end sequencing, therefore, I think, the expression level should be measured in FPKM rather than RPKM. edgeR is a package designed for differential expression analysis of RNA-seq data and you applied edgeR package to normalize the RNA-seq and limma package to detect differentially expressed (DE) genes. Why not use edgeR to normalize data and detect DE genes?

13.  For the Gencode V25 annotation for the GRCh37, the link in your manuscripts cannot be accessed, I think the correct one should be ftp://ftp.ebi.ac.uk/pub/databases/gencode/Gencode_human/release_25/GRCh37_mapping/gencode.v25lift37.annotation.gtf.gz.

14.  In Figure S12B, glia also showed a weak negative correlation between gene expression and mCpH, so, in my view, you said "only neurons showed a negative correlation" is not correct.

Attachments:

**Reviewer 2**

**Are you able to assess all statistics in the manuscript, including the appropriateness of statistical tests used?**

No, I do not feel adequately qualified to assess the statistics.

**Comments to author:**

DNA methylation is a covalent modification that regulates gene expression and defines cell fates. Previous reports have shown that DNA methylation in the human cerebral cortex is dynamically regulated throughout the life span. More recently, dynamic changes of global DNA methylation was reported during mouse and human brain development, and display cell type specific patterns between neurons and glias.

Price et al studied DNA methylation in brain development by NeuN sorting of neurons/non-neurons from Infant (0-1 years); Child (1-10 years), Teen (11-17 years) and Adult (18+ years).  This study shows that DNA methylation changes most dramatically during the first five years of postnatal life. The authors went on and showed that local mCpG and mCpH levels are highly correlated. By comparing with RNA -Seq data from the same brains, this study shows that mCpH levels are associated with mRNA splicing events for hundreds of transcripts (not necessarily a direct regulation). Quite interestingly, dynamic DNA methylation are enriched for risk factors of neuropsychiatric diseases, agreeing with very recent studies on cell type-specific DNA methylation and schizophrenia risk.

The Introduction could be more thorough and accurate: The concept of extensive methylome reconfiguration during development from fetal to young adult was already known; DNA methylation profiling based on NeuN sorting has been repeatedly used in previous studies. Because neurons and glias show differential gene expression, cell type specific DNA methylation could also be inferred for these DE genes.

**Authors Response**

**Point-by-point responses to the reviewers’ comments:**

Response to Reviewers:

*The authors thank the reviewers and editor for their attention and interest in our work. We have made many adjustments to incorporate their feedback as elaborated below. Revisions in response to reviewer comments are highlighted in yellow in the manuscript.*

Reviewer #1:

This paper provides the DNA methylation landscape of human neocortical development, and authors performed functional and phenotype associated studies to indicate the functions of methylation. Especially, they indicated CpH methylation exclusively regulate transcripts and their splicing events. I think this work is interesting. Here are my comments.

All figures are too low resolution to recognize the numbers on the axis, especially the supplementary figures.

*The figures have been updated to be higher resolution with larger axes labels. The former Figure S14 (molecular function enrichment of genes including methylation-associated alternative exons) has also been converted to a table for clarity.*

In Table S1 and S2, the brain IDs are not matched for some samples, such as Br906 in S2 while Br0906 in S1.

*These correspond to the same ID in our database, and are thus matched. Historically, we had used 4 digit IDs that sometimes had a leading 0 - however this caused issues having the 0 dropped when reading data into tools like Microsoft Excel. However, we have now standardized the brain IDs and have made them consistent throughout the paper (with the leading 0).*

We know that SNPs at CpG / CpH will interfere with methylation calls (PMID: 28822795), and we usually need to remove the known SNPs when we process the WGBS data. However, it is absent in your processing pipeline. I am wondering how many SNPs in your 18.7 million CpGs and 58.1 million CpHs and how they affect your results? In addition, for the coverage threshold of CpG and CpH, why you set different values (3 for CpG and 5 for CpH)?

*Unlike microarray technologies like the Illumina 450k/EPIC (where any SNP can potentially affect hybridization chemistry, e.g. PMID: 23314698), only SNPs with C/T alleles can potentially confound the DNAm estimates. This is because the DNAm proportions are calculated as C / C+T by Bismark. We therefore assessed what fraction of CpGs and CpHs contain C/T SNPs and then assessed enrichment for the subset of sites that were associated with cell type, age, or their interaction. Overall, there seemed to be little influence of common C/T SNPs (i.e., minor allele frequency, MAF > 5%) in these samples. For example, at the CpG level, while ~38.8% of the 18.7M CpGs overlapped an annotated SNP in dbSNP144 (and the vast majority were C/T SNPs), only a small fraction (~5%, N=342706) were actually polymorphic in our samples via Illumina array-based genotyping data from regular, non-bisulfite-converted DNA. Furthermore, among the CpGs significantly associated with cell type, age, and their interaction, less than 1% contained polymorphic C/T SNPs, which was quite depleted compared to the rest of methylome. For DMR analyses, the smoothing across neighboring CpGs likely attenuates the potential effects of C/T SNP confounding, and indeed, in our sensitivity analyses for ethnicity effects among DMRs, there was little confounding (see Figure S19). This further supports the minimal role of C/T SNPs confounding our findings. We observed similar degrees of polymorphic SNPs at CpH DNAm levels as well, with approximately 4% of CpH sites containing C/T SNPs polymorphic in our samples.*

*With regard to the different coverage cutoffs for CpG versus CpH DNAm analyses, we tried to select data-driven values that removed noisy sites. We especially wanted to be conservative with the CpH analyses, given the potentially much larger number of tests and then lack of replication datasets that were available. Furthermore, while the cutoffs were a bit different, the average coverage for CpGs before and after data processing were relatively similar. The mean coverage levels across samples among these considered sites had similar distributions across CpGs (median: 13, IQR: 10.6-15.8) and CpHs (median: 11.7 , IQR: 10.3-13.3), so if anything the mean coverage was slightly higher for CpGs.*

In Table S3, why the number of CpGs in homogenate samples is almost 1.5 fold of that in Neuron/Glia/Fetal samples while the number of CpHs are same? Additionally, why no methylation level statistics of CpH in homogenate samples?

*This difference was due to the difference in analyses performed on these groups of samples. The 18,664,892 CpGs were the set that had a minimum coverage of 3 reads across all neuronal and non-neuronal (ie, glial) samples and were used to calculate the differentially methylated regions (DMRs) by cell type, age, and the cdDMRs (see the “Identifying CpG differentially methylated regions (DMRs)” section of Methods). Fetal samples were also filtered to the matching set of 18,664,892 CpGs because we called methylation features (ie, LMRs, UMRs, etc.) using these CpGs in neuronal, glial, and fetal samples. Homogenate samples were not used in DMR or DNA methylation feature identification and so were not filtered to the same set of CpGs. Instead, they were filtered to the set of CpGs with coverage in all 55 postnatal samples. Because calling CpH methylation is very computationally intensive and homogenate mCpH levels would not be used for any other analyses in the paper, we did not call mCpH in the homogenate samples. As mCpH is primarily neuronal, the proportions are likely to be very similar to the neuronal samples from the same cortical dissection.*

You did the enrichment analysis of Glia and Neuron specific DE genes on marker genes for neuronal and non-neuronal identity. How to select these marker genes?

*The marker genes were selected from a previous publication, namely the cell type transcriptome database created by Zhang, Chen, Sloan, and colleagues (Journal Neuroscience, 2014). The “Differential expression between cell types” section of the methods is now updated to include this reference.*

For Figure S2A, B, C, and D, in my opinion, the axis titles are not very clear since the number (labeled on the axis) are effect coefficients rather than actual age for the x-axis and cell type for the y-axis. And, how to explain no zero (or approximately zero) age effect coefficients of individual CpGs as measured in homogenate samples?

*These panels show the relationship between detectable age- and cell identity-related DNAm level changes at individual CpGs when measured in homogenate compared to more cell type-resolved NeuN-sorted samples. Only CpGs that were differentially methylated by age in homogenate samples at p<1x10-4 are depicted, hence the lack of homogenate age coefficients with values at or near zero in these plots. Because some of the plots compare age effects after adjusting for cell type to unadjusted age estimates, we believe coefficients are an appropriate way to show how cell type changes mask age changes when measured in homogenate samples. To make the plots clearer to understand however, we have now listed the linear models in the figure and depicted which coefficient from the model is being measured on the x and y axes, as well as listing the number of CpGs in each quadrant.*

To clearly show LMRs and UMRs in different samples, could you make a supplementary table to show the number of LMRs and UMRs you detected? You mentioned, "Compared to prenatal homogenate cortex and postnatal glial cells, postnatal neurons showed a general accumulation of mCpG, at a rate 50% faster than the other cells." In section "DNAm as a map of putative functional genomic states". However, from Figure S3A and B, it is hard to get "50% faster", can you show it clearly? From Figure S3C, UMR and LMR showed similar enrichment in both adult and fetal brain, why you just emphasize LMR in manuscripts?

*The number of UMRs and LMRs identified in each sample are now reported in Supplementary Table 4. In terms of the rate of increasing methylation levels, neurons generally showed a faster global increase in methylation levels than glial or homogenate prenatal samples (~50% greater), and this is not visualized in the paper. However, greater increase in methylation of CpGs is also reflected in the decreasing number of neuronal low methylation regions (LMRs) and the percent of the genome they cover as reflected in Fig. S3A and S3B. The decreasing number and size of LMRs means that methylation is increasing in these regions; indeed, the slope of the linear model for decreasing neuronal LMR number and size in Fig. S3A and S3B are 5.5x and 8.7x that of the glial samples, respectively. These numbers are now reflected in the figure legend of Fig. S3.*

*We focused on the LMRs in the manuscript because LMRs are associated with cis-regulatory sequences which are known often to be cell type-specific and dynamic across development. Unmethylated regions (UMRs) primarily overlap promoters, and as many promoters include methylation-resistant CpG islands, UMR status is not as informative as gene body or LMR methylation for making inferences about gene or regulatory element activity. Because many promoters will be unmethylated regardless of the activity of the gene, we focused on LMRs instead.*

For Figure S5B, most of DMV associated TF genes (189) are overlapped across all four age groups, it seems that DMV overlapped TF genes in an age-independent manner rather than an age-dependent manner. How to explain this? For Figure S5C, the color Glia and Neurons are different from other Figures (like Figure S4C), please make it consistency.

*The color is now corrected in Figure S5C. As for the fact that most (189) of the TF genes overlapping the DNA methylation valleys we identified are shared, this reflects DMV biology and the fact that they are established early during differentiation, which occurs at an earlier time point than measured in this paper. Previous work has shown DMVs to be highly overlapping across fetal and adult cells (Xie et al, Cell, 2013) and that TF genes escaping DMVs can shed light on the developmental path of the cell type (Mo et al, Neuron, 2015). We have updated the language in this section to more precisely reflect these concepts.*

For Figure 1A, we can clearly see the methylation pattern of Infant Neuron is significantly different from others, where infant is defined as 0-1 years. For Figure S6C and D, we can see the range of coefficients in 0-6 years samples are larger than that in 6+ years samples in both glia and neuron. Based on these results, I think, it is hard to get the conclusion of "largest changes occurring in the first five years". In my opinion, you should make a similar analysis like Figure S6C and D for other age segments, like 0-1 years, 0-2 years, to clearly show the methylation changes during development.

*We based the conclusion that the largest shifts in DNAm occur within the first five years on the timing of the inflection point seen in the rate of change in the methylation levels in both CpG and CpH contexts. For instance, this can be seen in Figure S11 which shows the mean methylation levels in the six groups of cdDMRs. The methylation levels rapidly diverge between the two cell types between ages 0-5 years, then the slope becomes more flat. This can also be seen in Figure 2A, where CpH methylation generally accumulates steeply from ages 0-5 years, then less steeply for the remainder of years measured. Given the sparsity of samples between 1-5 years, it is hard to refine the developmental shifts beyond this conclusion, but subsetting to 0-3 years shows an even more pronounced difference as seen below.*

For Supplementary Table 5 the CpH context, how to calculate the "%Significant"? Because 7,682,075 / 58,100,000 = 13.22%, 3,194,618 / 58,100,000 = 5.50%, and 4,020,371 / 58,100,000 = 6.92% are different from what you showed in the table, where 58,100,000 is the number of CpH (Supplementary Table 3). You mentioned in manuscript "98.9% of 7,682,075 differentially methylated CpHs were in neurons, at FDR < 5%". I'm wondering if that means 7,597,572 (7 682 075 * 0.989) CpHs show hypomethylated in neurons? Please state it clearly in the manuscript.

*For assessing differential methylation at CpHs, we further filtered the ~58 million sites that had at least 5 coverage in all 32 NeuN-sorted WGBS samples to the 40,818,742 sites where at least 5 of the 32 samples had greater than 0% methylation (see the “Identification of differentially methylated positions” section of the methods). So the denominator is actually 40,818,742 CpH sites: 7,682,075 / 40,818,742 = 18.8%; 3,194,618 / 40,818,742 = 7.8%; and 4,020,371 / 40,818,742 = 9.8%. The text has now been clarified to state that 98.9% of the CpHs differentially methylated between neurons and glia at FDR<0.05 are hypermethylated in neurons (see line 207).*

I cannot find any information to indicate "first five years of postnatal life—similarly to mCpG—followed by a tapered global increase into adulthood" from Figure S10B. Both Figure S10C and Figure 2A show the proportions of mCpH changes with age increasing, however, the results are totally different and the statements in manuscript and figure legends are different. Please explain why. Figure S10D show 1,124 decreasing mCAG genes, 280 decreasing mCAC genes, and 1,750 increasing mCAC genes, however, in manuscript, you said "3,286 and 1,744 genes that contained significantly increasing and decreasing mCAC versus mCAG over development, respectively", why they are different?

*Figure S10B shows that the majority of differentially methylated CpH sites across age in neurons are in the CAC or CAG context and was meant to speak more to the first part of that sentence, namely that “most mCpH accumulated primarily in either the CAG or CAC context.” Figure S10C shows the early accumulation of methylation in the first five years and has now been added as evidence for that statement (see line 211).*

Both Figure S10C and Figure 2A show accumulation of mCpH in the CAC and CAG contexts, however while Figure S10C shows the overall proportion of (mCAC>10%)/(total CpH sites) or (mCAG>10%)/(total CpH sites), Figure 2A is normalized based on the number of CAG or CAC sites so the y-axis is instead (mCAC>10%)/(total CAC sites) or (mCAG>10%)/(total CAG sites). While this was mentioned in the text it was not at all clear in the figure legends, so the legends for both figures are now updated to be more explicit.

*The difference in the number of genes in Figure 10D compared to the manuscript text is because the biological process ontology analysis in Figure 10D was limited to genes overlapping significantly differentially methylated sites in neurons by age in either the CAC context or CAG context but not both. For instance, the “Decreasing mCAG” genes are those that do not also contain significantly decreasing mCAC. This distinction is what was intended by “exclusively overlapping CAG and CAC sites” in the legend but we recognize that this language could be more clear and have made an effort to make it so.*

The RNA-seq data was pair-end sequencing, therefore, I think, the expression level should be measured in FPKM rather than RPKM. edgeR is a package designed for differential expression analysis of RNA-seq data and you applied edgeR package to normalize the RNA-seq and limma package to detect differentially expressed (DE) genes. Why not use edgeR to normalize data and detect DE genes?

*We used limma to detect differential expression to be consistent with the differential methylation analysis methods (which also used limma-based empirical Bayes modeling) and also based on results in the voom paper that suggested that Poisson- or negative binomial-based modeling can be overly conservative (PMID: 24485249). Note that these two approaches had a high degree of authorship overlap (ie Gordon Symth as last author on both papers), and there has which has recently published combined workflows (PMID: 27441086).*

*We indeed used paired-end read counting with featureCounts and have therefore updated our acronym to FPKM throughout the paper to better fit with convention. We do note that RPKM and FPKM can produce identical values, depending on the exact normalization factor in the denominator, i.e. whether the "M" represents total reads mapped or total fragments mapped or assigned to genes, in millions. If M represents total fragments mapped or assigned to genes, each fragment counts as 1 in the numerator and 1 in the M(illion) normalizing denominator, whereas each pair of reads in single-ended RPKM count as 2 in the numerator and 2 in the denominator, thereby producing the same number.*

For the Gencode V25 annotation for the GRCh37, the link in your manuscripts cannot be accessed, I think the correct one should be ftp://ftp.ebi.ac.uk/pub/databases/gencode/Gencode_human/release_25/GRCh37_mapping/gencode.v25lift37.annotation.gtf.gz.

*The correct link is now included in the manuscript.*

In Figure S12B, glia also showed a weak negative correlation between gene expression and mCpH, so, in my view, you said "only neurons showed a negative correlation" is not correct.

*We have updated the text to reflect the weak negative correlation seen in glial samples.*

Reviewer #2:

DNA methylation is a covalent modification that regulates gene expression and defines cell fates. Previous reports have shown that DNA methylation in the human cerebral cortex is dynamically regulated throughout the lifespan. More recently, dynamic changes of global DNA methylation was reported during mouse and human brain development, and display cell type specific patterns between neurons and glias.

Price et al studied DNA methylation in brain development by NeuN sorting of neurons/non-neurons from Infant (0-1 years); Child (1-10 years), Teen (11-17 years) and Adult (18+ years). This study shows that DNA methylation changes most dramatically during the first five years of postnatal life. The authors went on and showed that local mCpG and mCpH levels are highly correlated. By comparing with RNA -Seq data from the same brains, this study shows that mCpH levels are associated with mRNA splicing events for hundreds of transcripts (not necessarily a direct regulation). Quite interestingly, dynamic DNA methylation are enriched for risk factors of neuropsychiatric diseases, agreeing with very recent studies on cell type-specific DNA methylation and schizophrenia risk.

The Introduction could be more thorough and accurate: The concept of extensive methylome reconfiguration during development from fetal to young adult was already known; DNA methylation profiling based on NeuN sorting has been repeatedly used in previous studies. Because neurons and glias show differential gene expression, cell type specific DNA methylation could also be inferred for these DE genes.

*We have updated the introduction to incorporate this feedback.*

**Second round of review**

**Reviewer 1**

Most of my questions have been addressed. I have another three minor comments:

1.      I think the author should summarize the influences of SNP on the CpG methylation and CpH methylation in “Discussion” section.

2.      The author said the median coverage of CpGs is 13 and the median coverage of CpHs is 11.7. And I cannot clearly understand why the threshold of CpG is 3, which is smaller than 5 (coverage threshold of CpH).

3.      For the answer of NO.9 question (the first revision), authors explained the conclusion of “largest changes occurring in the first five years” using Figure S11 and Figure 2A as evidence. I agree with this explanation. However, they did not revise the text (line 156 - line 158) in manuscripts. I think the author should add this evidence to the corresponding sentences and reorder the figures to make readers more clearly of this conclusion.
